# HDAC11 interacts with the NuRD (MTA3) complex to transcriptionally suppress TGFβ1 expression and inhibit hepatocellular carcinoma metastasis

**DOI:** 10.1186/s13148-026-02050-y

**Published:** 2026-01-17

**Authors:** Yang Yang, Jiaoli Wang, Qingqing Wu, Yishan Wang, Hui Meng, Lulu Zeng, Tian Qiu, Haixia Zhao, Qin Hu, Qiaoyou Weng, Meiling Liu, Minjiang Chen, Rongfang Qiu, Jiansong Ji, Weiqian Chen

**Affiliations:** 1 Zhejiang Key Laboratory of Imaging and Interventional Medicine, The Fifth Affliated Hospital of Wenzhou Medical University, Lishui City, 323000 Zhejiang Province China; 2 Cixi Biomedical Research Institute, Wenzhou Medical University, Ningbo City, 315000 Zhejiang Province China; 3 Department of Radiology, Lishui Central Hospital, Lishui City, 323000 Zhejiang Province China; 4 International Clinical School, Hangzhou Medical College, Lishui City, 323000 Zhejiang Province China; 5 Key Laboratory of Precision Medicine of Lishui, Lishui City, 323000 Zhejiang Province China; 6 Clinical College of The Affiliated Central Hospital, School of Medcine, Lishui University, Lishui City, 323000 Zhejiang Province China

**Keywords:** Hepatocellular carcinoma, HDAC11, MTA3, EMT, Metastasis

## Abstract

Hepatocellular carcinoma (HCC) is a leading global health concern, recognized for its complex pathogenesis and high mortality rates. The metastatic progression of HCC, considered the terminal event in tumor development, plays a pivotal role in determining patient prognosis, with metastasis being a key factor in poor survival outcomes.HDAC11 was found to be highly expressed in HCC tissues, with its elevated expression significantly correlating with poor patient survival. Both in vitro and in vivo experiments demonstrated that silencing HDAC11 led to a marked reduction in HCC cell proliferation. Interestingly, HDAC11 knockdown also resulted in a substantial increase in the metastatic potential of HCC cells. Mass spectrometry analysis revealed that HDAC11 interacts with the NuRD (MTA3) complex. Consistently, immunoprecipitation and GST pull-down assays demonstrated that the N-terminal region of HDAC11 directly binds to MTA3. Moreover, transcriptomic analysis indicated that HDAC11 represses *TGFB1* transcription, thereby inhibiting HCC metastasis. The enhanced metastatic phenotype induced by HDAC11 silencing was reversed upon concurrent down-regulation of TGFB1. Moreover, nanoparticles encapsulating both HDAC11 and TGF-β1 inhibitors effectively suppressed HCC cell proliferation and metastasis. This research elucidates the molecular mechanism by which HDAC11 inhibits metastasis and provides an effective strategy to mitigate the side effects associated with HDAC11 inhibition, offering novel insights and approaches for the precision treatment of HCC.

**Supplementary Information:**

The online version contains supplementary material available at 10.1186/s13148-026-02050-y.

## Introduction

Hepatocellular carcinoma (HCC) is the most common malignant liver tumor worldwide and ranks as the third leading cause of cancer-related deaths globally [1]. Due to the lack of obvious symptoms in the early stages, approximately 70% of patients are diagnosed at advanced stages, resulting in a low five-year survival rate of only about 18% [2]. Moreover, HCC metastasis is one of the primary causes of poor prognosis in HCC patients [3]. Metastasis is a complex, multi-step process that involves cancer cell invasion, detachment, circulation, colonization, and distant spread [4]. Metastatic HCC is typically diagnosed at advanced stages, resulting in difficult treatment and poor prognosis [5]. The metastasis and recurrence of HCC have become the most significant obstacles affecting the efficacy of HCC treatment and long-term prognosis [6–8]. Therefore, there is an urgent need for a deeper understanding of the molecular mechanisms underlying HCC metastasis in order to develop more effective therapeutic strategies.

Mammalian histone deacetylases (HDACs) consist of 18 members, classified into four groups (I-IV): class I (HDAC1, 2, 3, and 8), class II (HDAC4, 5, 6, 7, 9, and 10), class III (Sirtuins), and class IV (which includes only HDAC11) [9]. HDAC11 was first identified in 2002 and is the smallest member of the HDAC family. Its sequence is highly conserved across eukaryotic evolution. The catalytic core region of HDAC11 contains all of the conserved active site residues found in eukaryotic HDAC proteins [10]. HDAC11 exhibits deacetylase activity, removing acetyl groups from both histone and non-histone proteins. For example, HDAC11 regulates immune activation and tolerance in antigen-presenting cells (APCs) by modulating the acetylation levels of histones H3/H4 at the *IL-10* promoter [11]. In breast cancer, HDAC11 deacetylates and binds to E2Fa/E2F4, thereby suppressing the expression of the tumor suppressor gene ARH1 [12]. However, the role and molecular mechanisms of HDAC11 in HCC metastasis remain largely unexplored.

Here, we explored the impact of HDAC11 inhibition on HCC cells, revealing that while HDAC11 knockdown significantly inhibits cell proliferation, it paradoxically promotes HCC cell metastasis, an important and unexpected consequence that warrants further investigation. Mechanistically, we found that HDAC11 suppresses *TGFB1* expression by binding to the NuRD (MTA3) complex, thereby inhibiting HCC cell metastasis. To mitigate the side effects of HDAC11 inhibitors in HCC treatment, we utilized nanoparticles encapsulating both HDAC11 and TGF-β1 inhibitors. These nanoparticles effectively reduced the metastatic phenotype induced by HDAC11 inhibition, concurrently suppressing HCC cell proliferation and metastasis. Our findings highlight the importance of considering the potential metastatic side effects of HDAC11 inhibition in HCC therapy, emphasizing the need for a more precise and targeted approach to HCC treatment.

## Result

### Knockdown of HDAC11 inhibits the proliferation of HCC cells

**Fig. 1:**
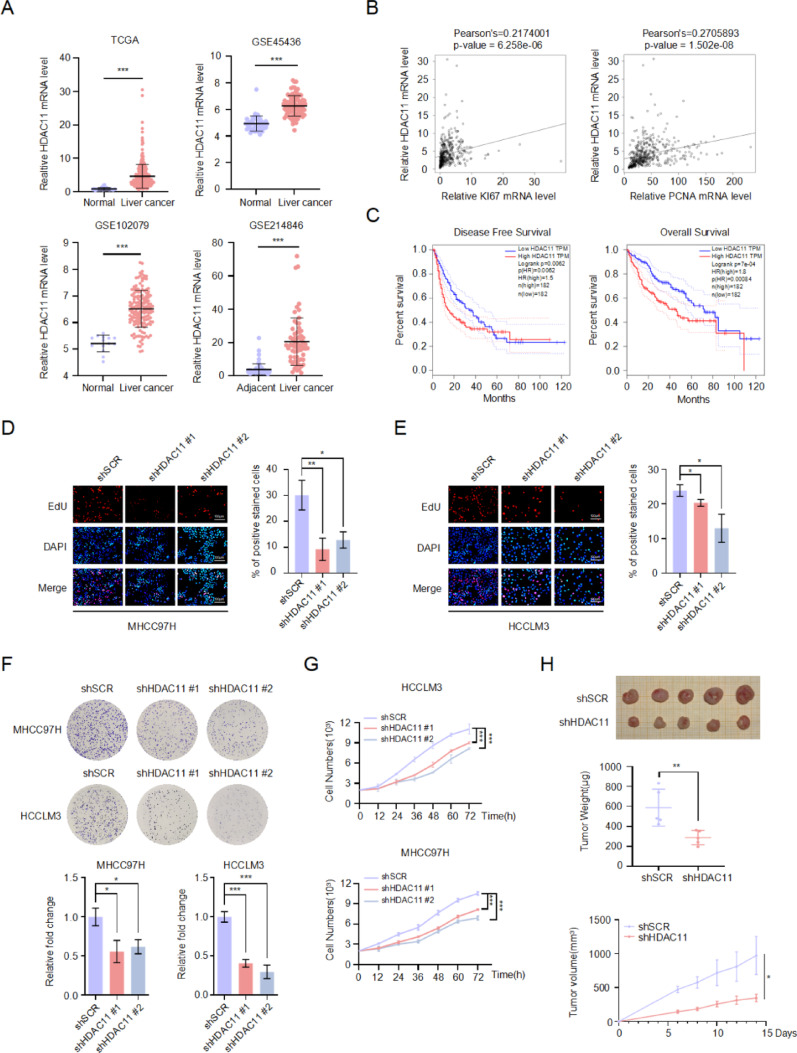
Expression of HDAC11 in liver cancer tissues and its role in promoting liver cancer cell proliferation. **A** Expression levels of HDAC11 in liver cancer and normal or adjacent liver tissues were analyzed using the TCGA, GSE45436, GSE102079, and GSE214846 databases. **B** The correlation between HDAC11 expression and that of KI67 and PCNA was assessed using the TCGA database. **C** The significance of HDAC11 expression in relation to overall survival (OS) and disease-free survival (DFS) in liver cancer patients was analyzed using the online GEPIA tool (http://gepia.cancer-pku.cn/) with data from the TCGA database. **D** In MHCC97H cells, the effect of HDAC11 knockdown on the proportion of EdU-positive cells was assessed by EdU incorporation assay, with red indicating EdU-positive cells and blue representing DAPI staining. **E** The effect of HDAC11 knockdown on EdU-positive cell percentage in HCCLM3 cells was assessed similarly, with red representing EdU-positive cells and blue representing DAPI staining. **F** Colony formation assays were performed to measure the number of colonies formed by MHCC97H and HCCLM3 cells following stable knockdown of HDAC11 compared to controls. **G** Cell growth curves were generated by monitoring the growth of HDAC11 knockdown and control cells at multiple time points. **H** Tumor growth was assessed in nude mice subcutaneously inoculated with stable HDAC11 knockdown or control HCCLM3 cells. Tumor size was measured every two days starting from day six, and after 14 days, tumors were excised and weighed for statistical analysis

To investigate the role of HDAC11 in hepatocellular carcinoma (HCC), we analyzed its expression levels in HCC and adjacent normal liver tissues using data from The Cancer Genome Atlas (TCGA) and Gene Expression Omnibus (GEO) databases. The results indicated that HDAC11 is significantly over-expressed in HCC tissues compared to adjacent normal tissues (Fig. 1A). Correlation analysis from the TCGA database revealed a significant positive correlation between HDAC11 expression and the levels of proliferation-related markers such as KI67 and PCNA (Fig. 1B). Prognostic analysis showed that higher HDAC11 expression is associated with poorer overall survival (OS) and disease-free survival (DFS) in HCC patients (Fig. 1C).

To validate the role of HDAC11 in HCC cell proliferation, we infected MHCC97H and HCCLM3 cells with shSCR (scramble-shRNA) and shHDAC11 viruses. EdU assays demonstrated that knockdown of HDAC11 significantly reduced the EdU-positive rate compared to control cells (Fig. 1D, E). Colony formation assays showed a significant decrease in colony formation in HDAC11 knockdown cells compared to controls (Fig. 1F). Growth curve experiments indicated that HDAC11 knockdown markedly slowed cell growth compared to the control group (Fig. 1G). Additionally, we subcutaneously injected stable HDAC11 knockdown HCCLM3 cells and control cells into nude mice. After 14 days, tumors were excised, and the results showed that knockdown of HDAC11 significantly inhibited subcutaneous tumor formation in nude mice (Fig. 1H). These findings suggest that HDAC11 plays a crucial role in promoting HCC cell proliferation.

### Knockdown of HDAC11 promotes the metastasis of HCC cells

**Fig. 2:**
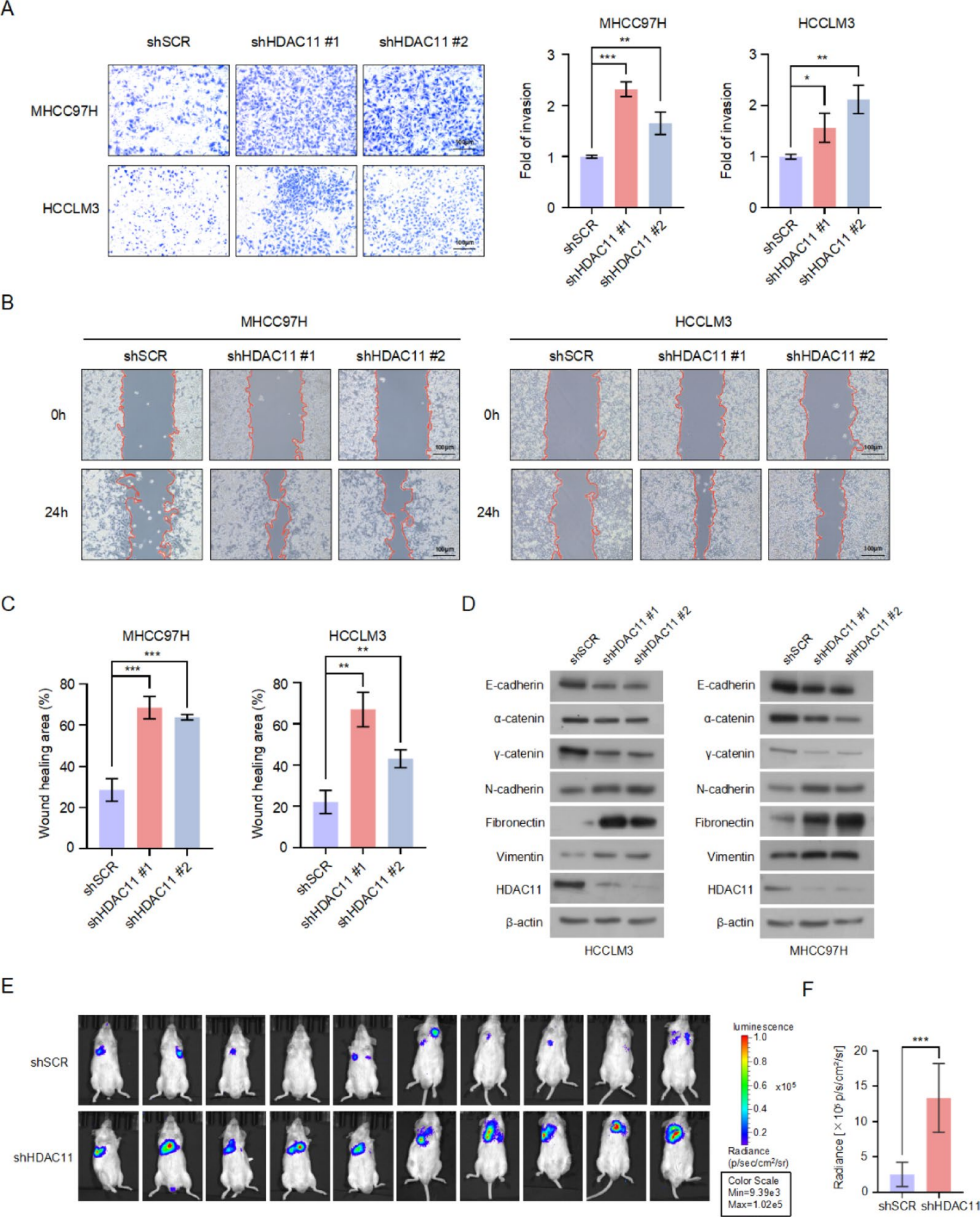
HDAC11 knockdown enhances liver cancer cell metastatic potential. **A** Invasion ability of MHCC97H and HCCLM3 cells with HDAC11 knockdown was evaluated using Transwell assays through Matrigel-coated invasion chambers, with results statistically analyzed. **B** Wound healing assays were performed on MHCC97H and HCCLM3 cells to assess the effect of HDAC11 knockdown on cell migration. **C** Statistical analysis of the wound healing area from panel B. **D** Western blot analysis was performed to assess the expression levels of epithelial markers (E-cadherin, α-catenin, γ-catenin) and mesenchymal markers (N-cadherin, fibronectin, vimentin) in HDAC11 knockdown and control cells. **E** Luminescence imaging of lung metastases in NOD-SCID mice intravenously injected with luciferase-expressing control or stable HDAC11 knockdown HCCLM3 cells. **F** Quantification of lung metastasis in mice from panel E

To investigate the role of HDAC11 in hepatocellular carcinoma (HCC) metastasis, we conducted transwell and wound healing assays on HDAC11 knockdown cells and control cells. The transwell assay results indicated that, compared to control cells, knockdown of HDAC11 significantly enhanced the invasive capacity of MHCC97H and HCCLM3 cells (Fig. 2A). Wound healing assays demonstrated that, following HDAC11 knockdown, HCC cells exhibited a significantly accelerated wound closure rate compared to control cells (Fig. 2B, C). These findings suggest that knockdown of HDAC11 enhances the invasion and migration abilities of HCC cells, indicating that HDAC11 may have a suppressive effect on metastasis, which is contrary to our initial expectations. Given that epithelial-mesenchymal transition (EMT) plays a crucial role in tumor cell metastasis, we further examined the expression of EMT-related markers in HDAC11 konckdown cells and control cells. The results showed that, after HDAC11 knockdown, the expression of epithelial markers E-cadherin, α-catenin, and γ-catenin was significantly reduced, while the expression of mesenchymal markers N-cadherin, fibronectin, and vimentin was significantly increased (Fig. 2D). These observations suggest that knockdown of HDAC11 promotes the progression of EMT, which aligns with the enhanced invasion and migration results. Subsequently, we conducted in vivo experiments to further assess the role of HDAC11 in metastasis. Stable HDAC11 knockdown and control HCCLM3 cells, labeled with a luciferase reporter gene, were injected into NOD-SCID mice via the tail vein. Two weeks later, lung metastasis was evaluated using small animal in vivo imaging systems. The results indicated that, compared to the control group, knockdown of HDAC11 significantly enhanced lung metastasis in the mice (Fig. 2E, F, Supplementary Fig. 1A). These results demonstrate that, although knockdown of HDAC11 significantly inhibits the proliferation of HCC cells, it concurrently increases their metastatic potential.

### HDAC11 interacts with the NuRD (MTA3) complex

**Fig. 3:**
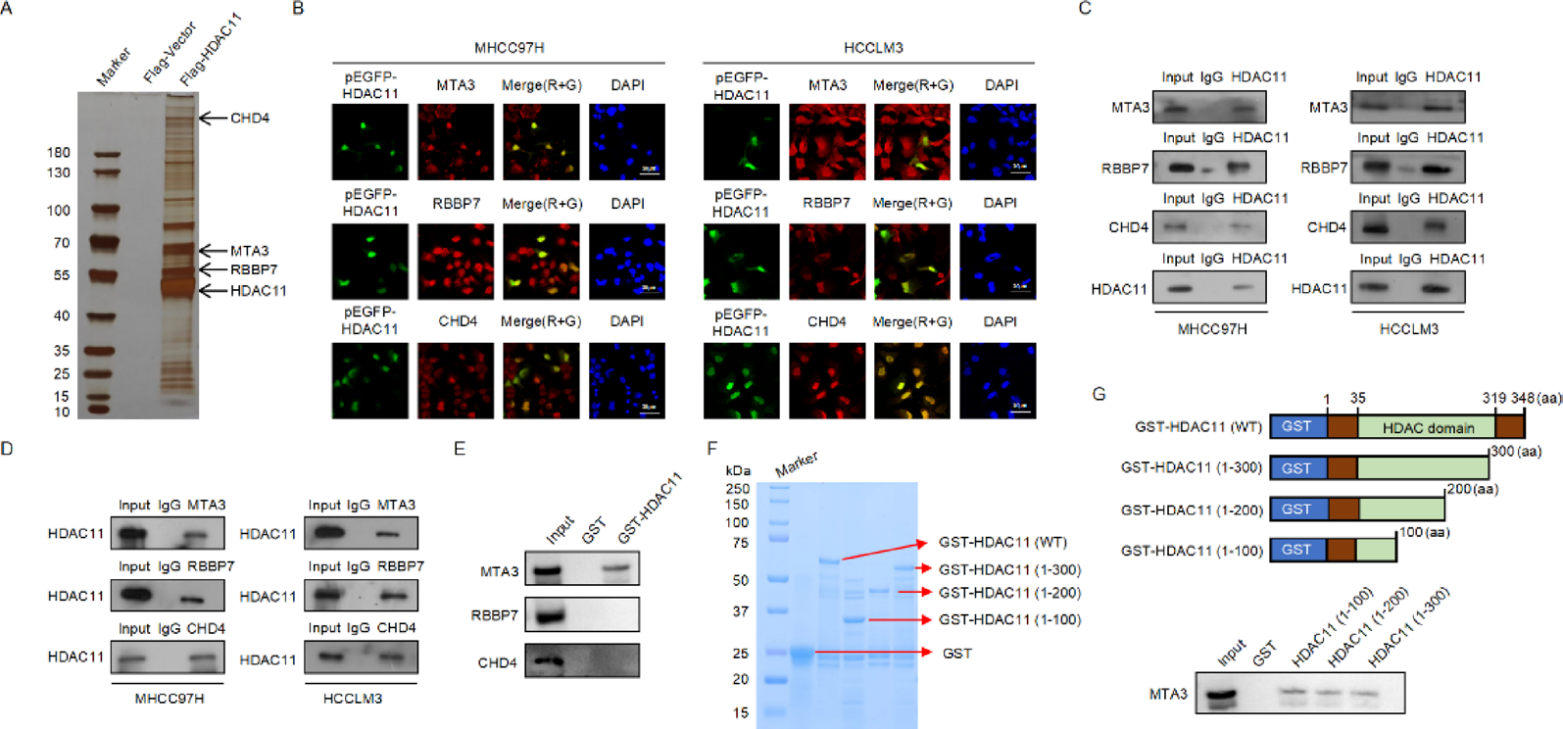
HDAC11 specifically interacts with the NuRD (MTA3) complex in vivo. **A** Silver-staining of FLAG-HDAC11 immunoprecipitates, followed by mass spectrometry to identify interacting proteins. Representative interacting proteins are annotated in the figure. **B** Immunofluorescence assays in MHCC97H and HCCLM3 cells transfected with pEGFP-HDAC11 to determine the cellular localization of HDAC11 and its interacting proteins. Green represents pEGFP-HDAC11 expression, red represents the interacting proteins, blue represents DAPI staining, and yellow represents co-localization of green and red signals. **C** Co-immunoprecipitation (IP) assays in MHCC97H and HCCLM3 cells were performed using antibodies against MTA3, RBBP7, or CHD4, followed by western blotting with anti-HDAC11 antibody to detect interactions between HDAC11 and these proteins. **D** IP assays in MHCC97H and HCCLM3 cells using anti-HDAC11 antibody, followed by western blotting with other antibodies to validate the binding of HDAC11 to its interacting partners. **E** GST pull-down assays were performed using bacterially expressed GST-HDAC11 protein and in vitro–transcribed/translated MTA3, RBBP7, and CHD4. **F** Coomassie blue staining of GST-HDAC11 and its truncated constructs purified from bacterial expression systems. **G** Schematic diagram of GST-HDAC11 truncation constructs and their interactions with MTA3 determined by GST pull-down assays

To investigate the molecular mechanisms by which HDAC11 regulates hepatocellular carcinoma (HCC) metastasis, we transfected Flag-HDAC11 vectors into MHCC97H cells and performed immunoprecipitation, silver staining, and mass spectrometry to identify HDAC11-interacting proteins. The mass spectrometry results revealed that HDAC11 specifically associates with core components of the NuRD (MTA3) complex, including CHD4, RBBP7, and MTA3 (Fig. 3A). Subsequent KEGG pathway analysis of these HDAC11-interacting proteins indicated significant associations with pathways such as Tight Junction, Nucleocytoplasmic Transport, Mitophagy, Gap Junction, Endocytosis, DNA Replication, Central Carbon Metabolism in Cancer, Cell Cycle, Autophagy, and the AMPK Signaling Pathway (Supplementary Fig. 1B). We transfected MHCC97H and HCCLM3 cells with EGFP-tagged pEGFP-HDAC11 vectors and performed immunofluorescence co-localization experiments using endogenous antibodies against MTA3, RBBP7, and CHD4. The results demonstrated co-localization of HDAC11 with MTA3, RBBP7, and CHD4 in the nucleus, further confirming the interaction between HDAC11 and the NuRD (MTA3) complex (Fig. 3B). To further validate these findings, we conducted co-immunoprecipitation and immunofluorescence co-localization experiments. In both MHCC97H and HCCLM3 cells, immunoprecipitation with antibodies against MTA3, RBBP7, and CHD4 followed by Western blotting with an anti-HDAC11 antibody confirmed the interaction between HDAC11 and these NuRD (MTA3) complex components (Fig. 3C). Conversely, immunoprecipitation with an anti-HDAC11 antibody and subsequent Western blotting with antibodies against MTA3, RBBP7, and CHD4 further corroborated these interactions (Fig. 3D). The NuRD complex comprises three distinct complexes: NuRD (MTA1), NuRD (MTA2), and NuRD (MTA3). While NuRD (MTA1) and NuRD (MTA2) complexes are associated with promoting tumor cell metastasis, the NuRD (MTA3) complex is known to suppress metastasis. Notably, our mass spectrometry analysis did not detect MTA1 or MTA2, but identified MTA3, suggesting that HDAC11 specifically associates with the NuRD (MTA3) complex to exert its suppressive effect on metastasis. To confirm the specificity of this interaction, we performed co-immunoprecipitation experiments to assess potential interactions between HDAC11 and MTA1 or MTA2. The results aligned with our expectations, showing no interaction between HDAC11 and MTA1 or MTA2, thereby supporting the specificity of the HDAC11-NuRD (MTA3) complex association (Supplementary Fig. 1C). To further delineate which components within the NuRD (MTA3) complex directly interact with HDAC11, we generated a GST-tagged HDAC11 construct and performed GST pull-down assays. The results demonstrated that HDAC11 directly interacted with MTA3 in vitro, whereas no direct interaction was detected between HDAC11 and either RBBP7 or CHD4 (Fig. 3E). To further map the specific domain of HDAC11 responsible for MTA3 binding, a series of truncated GST-HDAC11 constructs were generated, including GST-HDAC11 (1-100 aa), GST-HDAC11 (1-200 aa), and GST-HDAC11 (1-300 aa). Coomassie Brilliant Blue staining confirmed the successful in vitro purification of both the full-length and truncated forms of GST-HDAC11 (Fig. 3F). GST pull-down assays revealed that all three truncated proteins retained the ability to bind MTA3, indicating that the N-terminal region of HDAC11 (amino acids 1-100) is sufficient and essential for its interaction with MTA3 (Fig. 3G). Collectively, these findings indicate that HDAC11 specifically associates with the NuRD (MTA3) complex, providing insights into the molecular mechanisms underlying HDAC11’s role in suppressing HCC metastasis.

### Transcriptome sequencing analysis of HDAC11

**Fig. 4:**
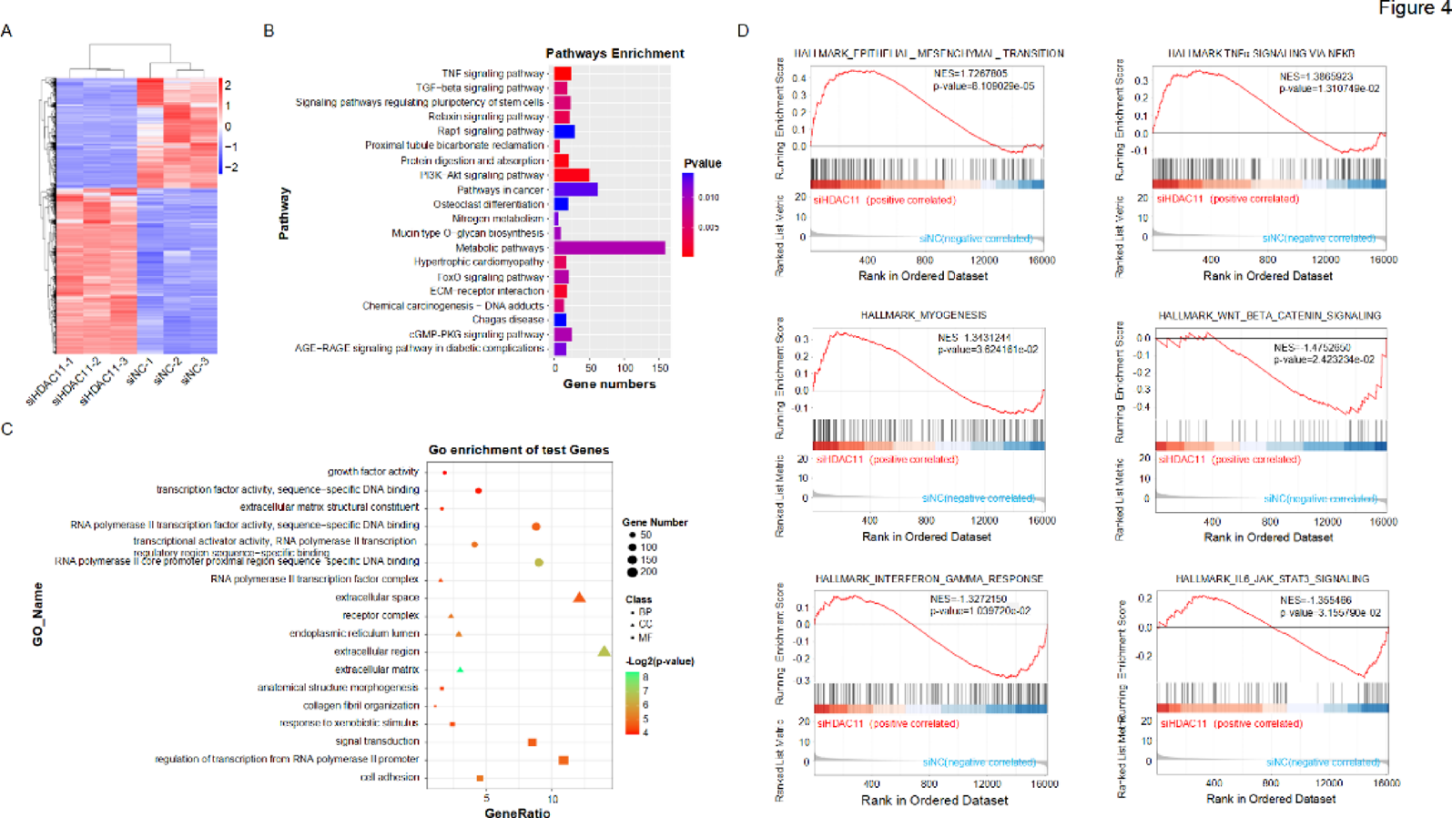
Transcriptomic analysis of HDAC11. **A** RNA sequencing was performed on control and HDAC11 knockdown cells, and differentially expressed genes (logFC ≥ 1 or ≤ -1, *p* < 0.05) were analyzed using heatmaps. **B** KEGG pathway analysis of the differentially expressed genes, displayed as a bar graph. **C** Gene ontology (GO) enrichment analysis of the differentially expressed genes, shown as a bubble plot. **D** Gene set enrichment analysis (GSEA) comparing the enrichment of hallmark pathways in HDAC11 knockdown versus control groups

To investigate the molecular mechanisms by which HDAC11 regulates hepatocellular carcinoma (HCC) metastasis, we transfected MHCC97 cells with HDAC11 siRNA and control siRNA. Subsequently, RNA sequencing (RNA-seq) was performed to analyze gene expression profiles. Heatmap analysis revealed that, compared to control cells, the expression of differentially expressed genes (DEGs) was predominantly upregulated upon HDAC11 knockdown, suggesting that HDAC11 plays a significant role in transcriptional repression (Fig. 4A). KEGG pathway analysis of these DEGs indicated significant associations with pathways such as the TNF signaling pathway, TGF-β signaling pathway, signaling pathways regulating pluripotency of stem cells, relaxin signaling pathway, and Rap1 signaling pathway (Fig. 4B). Gene Ontology (GO) analysis of the DEGs further revealed that, within the Biological Process (BP) category, HDAC11 target genes are enriched in processes including growth factor activity, transcription factor activity, sequence-specific DNA binding, and extracellular matrix structural constituent. In the Cellular Component (CC) category, these target genes are closely associated with the RNA polymerase II transcription factor complex, extracellular space, and receptor complex. Within the Molecular Function (MF) category, they are enriched in processes such as anatomical structure morphogenesis, collagen fibril organization, and response to xenobiotic stimulus (Fig. 4C). Gene Set Enrichment Analysis (GSEA) revealed significant enrichment of pathways such as epithelial–mesenchymal transition (EMT) and TNFα signaling via NF-kappaB in the HDAC11 knockdown group (Fig. 4D). These results further suggest that interference with HDAC11 may promote the occurrence of EMT and metastasis.

### HDAC11 transcriptionally represses the expression of *TGFB1*

**Fig. 5:**
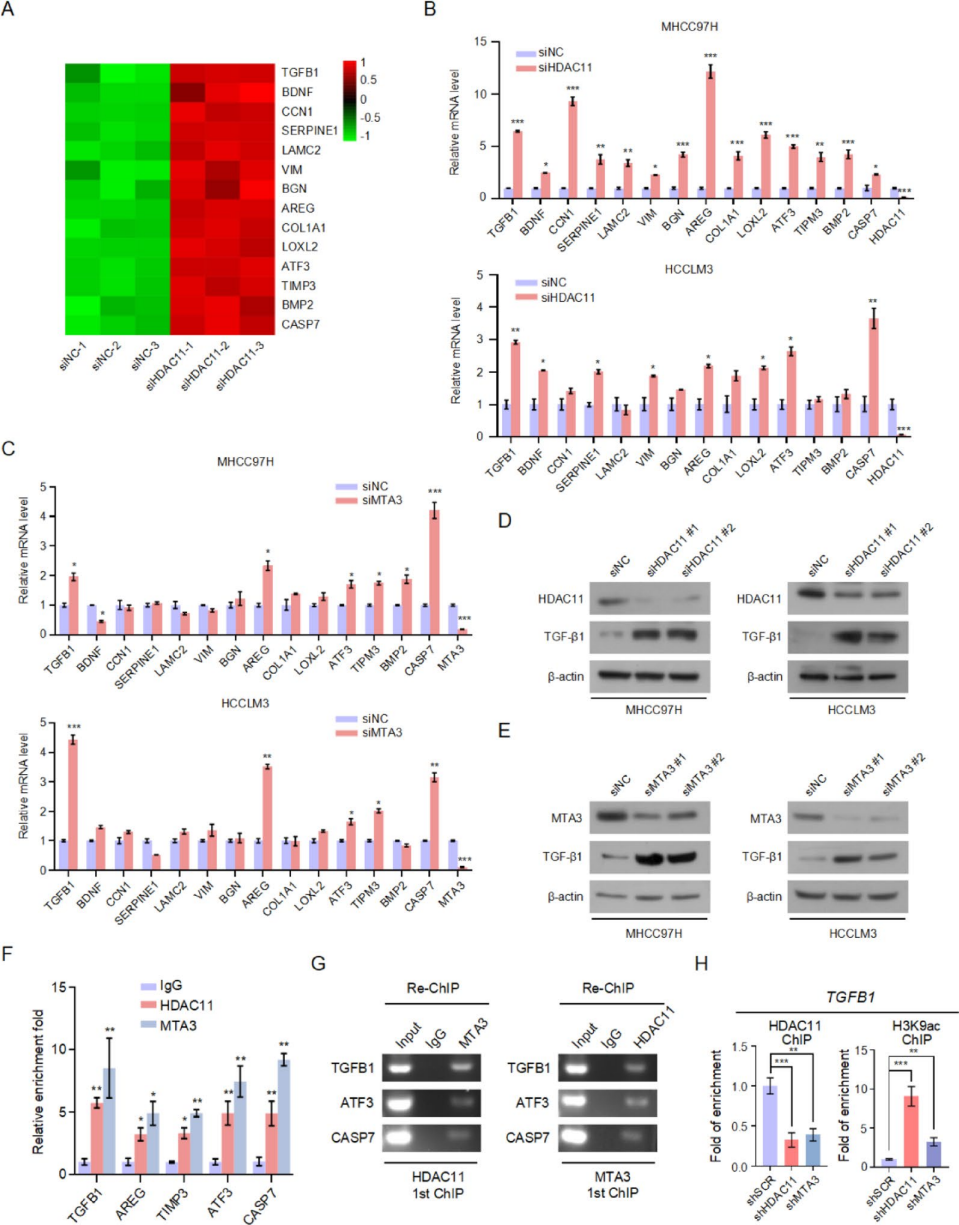
HDAC11/NuRD (MTA3) complex transcriptionally represses *TGFB1* expression. **A** Heatmap analysis of 14 representative target genes. **B** RT-PCR analysis of the mRNA expression levels of representative target genes following HDAC11 knockdown in MHCC97H and HCCLM3 cells compared to controls. **C** RT-PCR analysis of the mRNA expression levels of representative target genes following MTA3 knockdown in MHCC97H and HCCLM3 cells compared to controls. **D** Western blot analysis of TGFB1 expression levels in HDAC11 knockdown and control MHCC97H and HCCLM3 cells. **E** Western blot analysis of TGFB1 expression levels in MTA3 knockdown and control MHCC97H and HCCLM3 cells. **F** ChIP assays to assess the occupancy of HDAC11 and MTA3 at the promoter regions of target genes. IgG was used as a negative control. **G** ChIP-re-ChIP assays to investigate whether HDAC11 and MTA3 form a complex that co-occupies the promoters of *TGFB1*, *AREG1*, and *CASP7*. The first and second rounds of ChIP were performed using different antibodies as indicated. **H** qChIP assays were performed to evaluate the enrichment of HDAC11, MTA3, and histone H3K9ac at the *TGFB1* promoter in HCCLM3 cells upon knockdown of HDAC11 or MTA3

Given the role of HDAC11 and the NuRD (MTA3) complex in transcriptional repression, we selected 14 target genes from our RNA sequencing data that exhibited increased expression following HDAC11 interference (Fig. 5A). RT-PCR validation revealed that, compared to the control group, interference with HDAC11 significantly upregulated the expression levels of target genes including *TGFB1*,* BDNF*,* CCN1*,* SERPINE1*,* LAMC2*,* VIM*,* BGN*,* AREG*,* COX2*,* ATF3*,* TIMP3*,* BMP2*, and *CASP7* (Fig. 5B). Considering the interaction between HDAC11 and the NuRD (MTA3) complex, we examined the effects of MTA3 interference on the expression of HDAC11 target genes. Results indicated that MTA3 interference led to a significant increase in the expression levels of *TGFB1*, *AREG*, *ATF3*, *TIMP3*, and *CASP7*, consistent with the effects observed upon HDAC11 interference (Fig. 5C). Among these, TGFB1 is known to significantly enhance tumor cell metastatic potential and facilitate epithelial-mesenchymal transition (EMT), making it a key target in promoting tumor cell metastasis. Therefore, we selected *TGFB1* as the regulatory target gene of the HDAC11/NuRD (MTA3) complex. Western blot analysis demonstrated that interference with HDAC11 significantly increased TGFB1 expression, and similarly, MTA3 interference also induced TGFB1 expression (Fig. 5D, E). To verify whether HDAC11 and MTA3 directly bind to the promoters of target genes to participate in transcriptional regulation, we conducted chromatin immunoprecipitation (ChIP) experiments. The results showed that both HDAC11 and MTA3 directly occupy the promoters of target genes such as *TGFB1*, *AREG*, *ATF3*, *TIMP3*, and *CASP7*, indicating their involvement in direct transcriptional regulation (Fig. 5F). Furthermore, ChIP-re-ChIP experiments were performed to confirm whether HDAC11 and MTA3 function as a complex to co-regulate target gene expression. Initially, ChIP was performed using an HDAC11 antibody, followed by a second round of ChIP with an MTA3 antibody. PCR results revealed that after two rounds of ChIP, DNA fragments of target genes *TGFB1*, *AREG*, and *CASP7* were successfully amplified, suggesting that the HDAC11/MTA3 complex co-regulates the expression of these target genes (Fig. 5G). Additionally, qChIP assays revealed that HDAC11 knockdown significantly reduced the enrichment of both HDAC11 and MTA3 at the *TGFB1* promoter. Consistently, silencing of HDAC11 markedly increased the acetylation level of histone H3K9 at the *TGFB1* promoter region, whereas MTA3 knockdown also led to a partial increase in H3K9 acetylation at the same locus (Fig. 5H). These results indicate a functional coordination between HDAC11 and MTA3 in the epigenetic regulation of the target gene *TGFB1*.

**Fig. 6:**
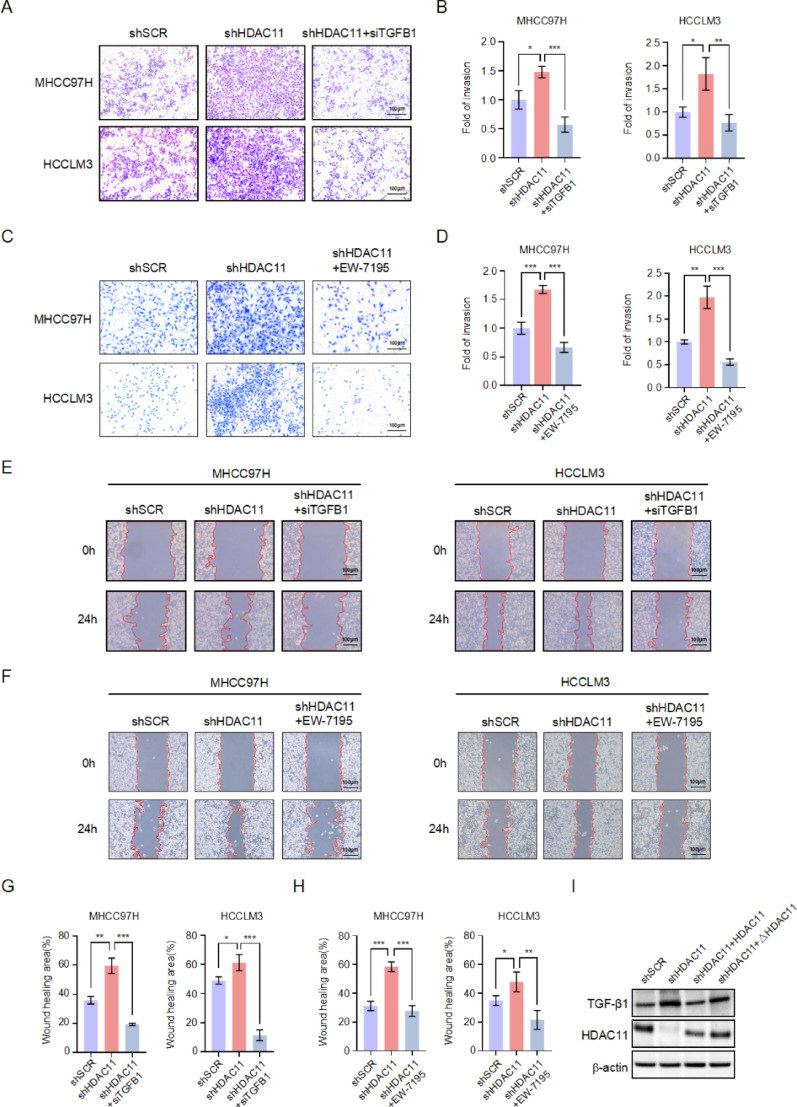
Increased liver cancer metastasis due to HDAC11 knockdown is inhibited by concurrent TGFB1 knockdown. **A** Invasion ability of MHCC97H and HCCLM3 cells was assessed using Transwell assays in control, HDAC11 knockdown, and HDAC11/TGFB1 double knockdown groups. **B** Statistical analysis of the number of cells invading through Matrigel-coated Transwell chambers from panel A. **C** Transwell assays to determine whether TGF-β1 inhibitor (EW-7195) can rescue the metastatic phenotype induced by HDAC11 knockdown in MHCC97H and HCCLM3 cells. **D** Quantification of cell invasion in panel C. **E** Wound healing assays to evaluate the effects of TGFB1 inhibition on the migration rate of MHCC97H and HCCLM3 cells in the control, HDAC11 knockdown, and HDAC11/TGFB1 double knockdown groups. **F** Wound healing assays to assess whether TGFB1 inhibition can rescue the migration increase induced by HDAC11 knockdown in MHCC97H and HCCLM3 cells. **G** and **H** Quantification of wound healing percentage from panels E and F, respectively. **I** Western blot analysis of TGFB1 expression under the indicated conditions. ΔHDAC11 represents the catalytically inactive HDAC11 mutant generated by deletion of the core enzymatic region spanning amino acids 129–157

To further investigate whether the suppression of metastasis induced by HDAC11 interference is due to its regulation of TGFB1, we simultaneously interfered with TGFB1 during HDAC11 knockdown. Transwell assays demonstrated that in MHCC97H and HCCLM3 cells, interference with HDAC11 significantly enhanced tumor cell invasion; however, simultaneous interference with TGFB1 reversed this effect, leading to a significant reduction in invasion (Fig. 6A, B). Additionally, the inclusion of a TGF-β1 inhibitor during HDAC11 knockdown also reversed the increased invasion phenotype induced by HDAC11 interference (Fig. 6C, D). Cell migration assays revealed that in MHCC97H and HCCLM3 cells, knockdown of HDAC11 significantly enhanced tumor cell migration; however, simultaneous knockdown of TGFB1 resulted in a significant decrease in migration (Fig. 6E, G). Similarly, the addition of a TGF-β1inhibitor during HDAC11 konckdown reversed the increased migration phenotype induced by HDAC11 interference (Fig. 6F, H). Additionally, to determine whether the regulatory effect of HDAC11 on the target gene TGFB1 depends on its deacetylase enzymatic activity, we generated an enzymatically inactive mutant of HDAC11 by deleting the critical catalytic region (Flag-HDAC11 **Δ**129–157). Upon HDAC11 knockdown, the expression of TGFB1 was markedly up-regulated. Re-expression of wild-type HDAC11 fully restored TGFB1 expression to basal levels. In contrast, reconstitution with the catalytically inactive **Δ**HDAC11 failed to rescue the elevated TGFB1 expression, which remained significantly increased (Fig. 6I). These results demonstrate that the suppressive effect of HDAC11 on TGFB1 transcription is dependent on its deacetylase enzymatic activity. These results collectively suggest that the increased cell metastatic potential observed upon HDAC11 knockdown is attributed to its negative regulation of TGFB1.

### Nanoparticles encapsulating HDAC11 and TGF-β1 inhibitors effectively suppress both the proliferation and metastasis of HCC cells.

**Fig. 7:**
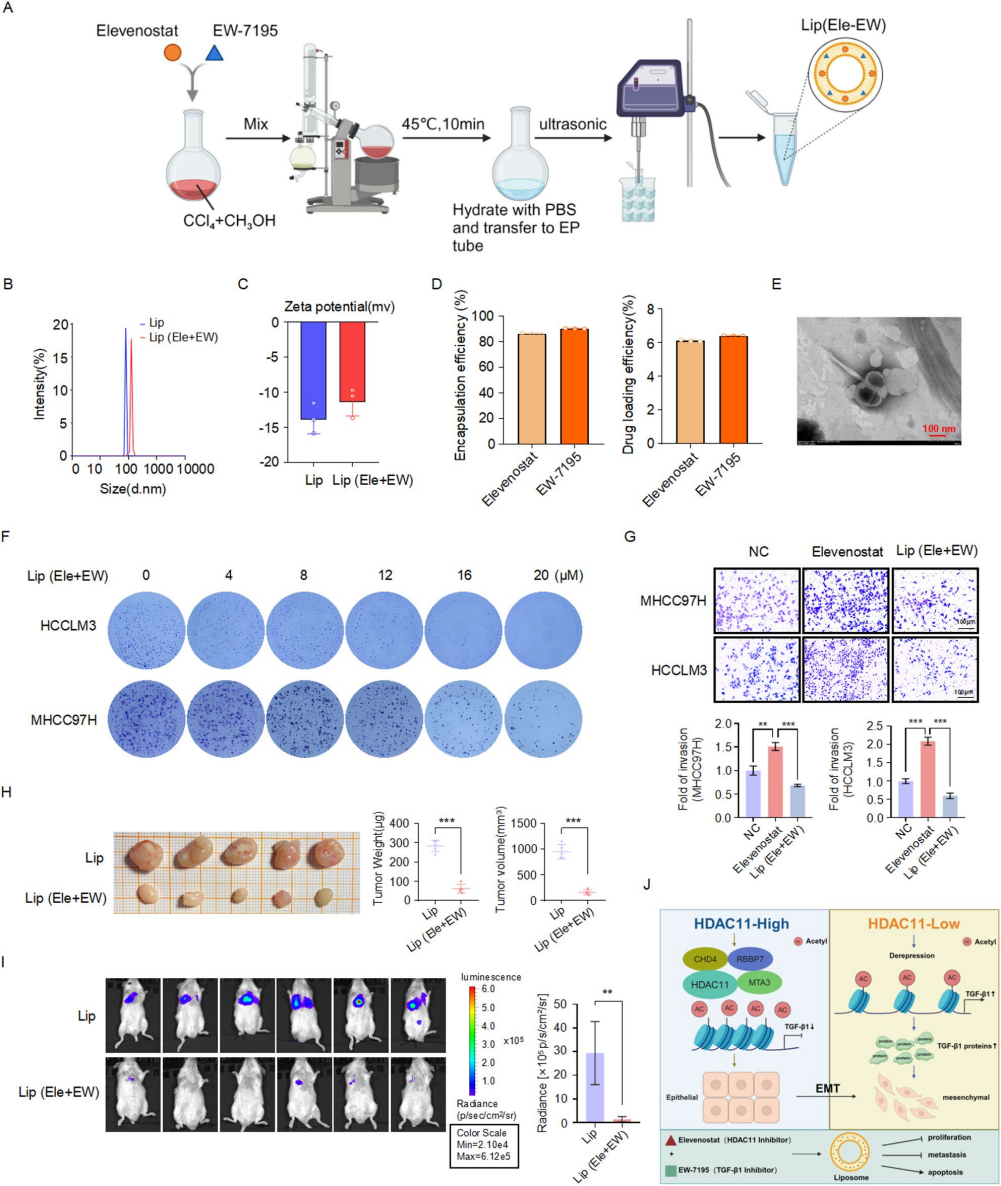
Liposomal nanoparticles encapsulating HDAC11 and TGF-β1 inhibitors suppress liver cancer proliferation and metastasis. **A** Schematic diagram of the preparation of liposomal nanoparticles encapsulating HDAC11 inhibitor (Elevenostat) and TGF-β1 inhibitor (EW-7195). **B** Measurement of the particle size of the synthesized liposomal nanoparticles Lip (Ele + EW). **C** Zeta potential measurement of Lip (Ele + EW) liposomal nanoparticles. **D** Encapsulation and loading efficiency of Elevenostat and EW-7195 in Lip (Ele + EW) nanoparticles. **E** Electron microscopy image of Lip (Ele + EW) liposomal nanoparticles. **F** Colony formation assays to evaluate the effect of different concentrations of Lip (Ele + EW) nanoparticles on liver cancer cell proliferation. **G** Transwell assays to assess the effect of Lip (Ele + EW) nanoparticles on liver cancer cell metastasis. **H** Inhibitory effects of Lip (Ele + EW) nanoparticles on subcutaneous xenograft tumors in mice. Drug administration was initiated on day 8 after tumor implantation, followed by dosing every two days for a total of four administrations. Mice were sacrificed two weeks later for measurement of tumor volume and tumor weight. Lip denotes the liposome control group. **I** Inhibitory effects of Lip (Ele + EW) nanoparticles on lung metastasis in mice. Drug administration was initiated three days after tail vein injection of tumor cells, followed by dosing every two days for a total of four administrations. Lung metastasis was monitored by in vivo bioluminescence imaging. Lip denotes the liposome control group. **J** Schematic model summarizing the findings of this study

The molecular mechanism by which HDAC11 regulates liver cancer metastasis has been clarified. To mitigate the side effects associated with increased metastasis caused by the use of HDAC11 inhibitors, we employed liposomal nanoparticles encapsulating both the HDAC11 inhibitor (Elevenostat) and the TGF-β1 inhibitor (EW-7195) (Fig. 7A). This approach effectively reduces the side effects of Elevenostat and enhances the delivery of the drugs into tumor cells. The synthesized material was named Lip (Ele + EW), and characterization of this nanomaterial was performed. Dynamic light scattering (DLS) measurements revealed that the average particle size of Lip (Ele + EW) nanoparticles was 125.63 ± 7.52 nm (Fig. 7B). The Zeta potential of Lip (Ele + EW) nanoparticles was − 11.29 ± 2.09 mV (Fig. 7C). The encapsulation efficiency of Elevenostat in Lip (Ele + EW) was 86.13 ± 0.61%, while the encapsulation efficiency of EW-7195 was 90.20 ± 0.20%. The drug loading rates of Elevenostat and EW-7195 in Lip (Ele + EW) nanoparticles were 6.10 ± 0.05% and 6.39 ± 0.15%, respectively (Fig. 7D). Transmission electron microscopy (TEM) analysis indicated that the average particle size of Lip (Ele + EW) nanoparticles was approximately 114.97 ± 6.83 nm (Fig. 7E). Treatment of HCCLM3 and MHCC97H cells with Lip (Ele + EW) nanoparticles demonstrated significant inhibition of liver cancer cell proliferation, as shown by colony formation assays (Fig. 7F). Furthermore, Transwell assays indicated that Lip (Ele + EW) nanoparticles effectively counteracted the metastatic phenotype induced by Elevenostat, which is associated with increased metastasis (Fig. 7G). To further validate the effects of Lip (Ele + EW) nanoparticles on tumor proliferation and metastasis in vivo, we established both a subcutaneous xenograft model and lung metastasis model. In the subcutaneous tumor model, drug administration was initiated by intraperitoneal injection on day 8 after tumor implantation, followed by dosing every two days for a total of four administrations. The results showed that, compared with the control group, treatment with Lip (Ele + EW) nanoparticles significantly suppressed both tumor volume and tumor weight (Fig. 7H). In the experimental lung metastasis model, tumor cells were injected via the tail vein, and intraperitoneal administration of Lip (Ele + EW) nanoparticles was initiated three days after cell injection, followed by dosing every two days for a total of four administrations. The results demonstrated that, compared with the control group, Lip (Ele + EW) nanoparticles treatment markedly reduced the extent of lung metastasis in mice (Fig. 7I). These results suggest that the combined targeting of HDAC11 and TGF-β1 can effectively suppress both the proliferation and metastasis of liver cancer cells.

## Discussion

Liver cancer metastasis is a hallmark of advanced liver cancer, leading to poor prognosis and significantly increasing mortality risks in patients. The molecular mechanisms underlying liver cancer metastasis are highly complex, involving epithelial-mesenchymal transition (EMT), extracellular matrix remodeling, angiogenesis and lymphangiogenesis, metabolic reprogramming, and immune-suppressive cells, among others [8]. In-depth research into the molecular mechanisms of liver cancer metastasis is crucial for identifying new therapeutic targets and optimizing personalized treatment strategies, thereby improving the survival rate of liver cancer patients. In this study, we found that HDAC11 promotes the proliferation of liver cancer cells but inhibits their metastasis. Specifically, HDAC11 can bind to the NuRD (MTA3) complex, but it does not interact with MTA1 and MTA2. The NuRD complex is a chromatin-remodeling complex that plays a key role in gene transcription regulation, cell differentiation, development, and tumorigenesis [13, 14]. The MTA (Metastasis-Associated) family members are important components of the NuRD complex, mediating its interaction with chromatin. MTA1, MTA2, and MTA3 are three isoforms of this family, each playing different roles in tumor metastasis, development, and cell metabolism [15]. Among them, MTA1 and MTA2 primarily interact with HDAC1 and HDAC2 to promote the EMT process, thus enhancing tumor cell metastasis [16, 17]. Conversely, MTA3 exerts an opposing effect, primarily inhibiting EMT and metastasis [18]. Our research demonstrates that HDAC11 specifically recruits the NuRD (MTA3) complex to suppress the expression of *TGFB1*, thereby inhibiting liver cancer cell metastasis. TGF-β1 (Transforming Growth Factor Beta 1) is a multifunctional cytokine that plays a crucial role in tumorigenesis, proliferation, metastasis, and immune evasion [19, 20]. In normal cells and early-stage tumors, TGF-β1 primarily inhibits cell proliferation by arresting the cell cycle. However, during tumor progression, particularly in advanced cancer stages, the function of TGF-β1 switches to promote tumor cell proliferation [20, 21]. Furthermore, TGF-β1 enhances tumor metastasis by inducing EMT, extracellular matrix degradation, angiogenesis, and resistance to anti-cancer treatment [22]. Through rescue experiments, we demonstrated that co-interference of HDAC11 and TGFB1 effectively suppresses the increased metastatic phenotype induced by HDAC11 interference. This suggests that the metastasis-promoting effect of HDAC11 interference is primarily due to the suppression of *TGFB1* expression by HDAC11 binding to the NuRD (MTA3) complex.

Currently reported HDAC11-selective inhibitors include FT895, Elevenostat, compound 5, TD034, SIS17, and Garcinol [23–27]. Existing studies have primarily focused on evaluating their inhibitory potency toward HDAC11 enzymatic activity. Only a limited number of reports have examined their effects on tumor cell proliferation, whereas their impact on tumor metastasis has largely remained unexplored. Our results demonstrate that inhibition of HDAC11 effectively suppresses hepatocellular carcinoma cell proliferation; however, the surviving tumor cells exhibit a markedly enhanced metastatic capacity. Notably, a previous study reported that HDAC11 inhibition promotes metastatic dissemination of murine breast cancer cells [28], which is highly consistent with our findings. Collectively, these observations raise an important safety concern that HDAC11-targeted therapy, particularly in the context of acquired drug resistance, may carry an unintended risk of promoting tumor metastasis. In terms of selectivity, several HDAC11 inhibitors have been reported to display excellent specificity. For instance, FT895 is a highly selective HDAC11 inhibitor, exhibiting more than 1,000-fold selectivity over other members of the HDAC family [23]. Nonetheless, certain HDAC11 inhibitors show measurable off-target activities. For example, compound 5 not only inhibits HDAC11 but also displays partial inhibitory effects toward HDAC6 and HDAC8 [25]. Similarly, Garcinol, in addition to targeting HDAC11, exerts inhibitory activity against HDAC8 [29]. Moreover, it has been reported that genetic silencing of HDAC1 in BT187 cells leads to a compensatory increase in the expression of HDAC10 and HDAC11 [30], indicating intrinsic plasticity within the HDAC regulatory network. However, to date, no study has systematically demonstrated whether inhibition of HDAC11 triggers compensatory upregulation or functional substitution by other HDAC family members. This potential compensatory mechanism remains to be elucidated and warrants further investigation using transcriptomic and proteomic profiling approaches.

Previous studies have indicated that HDAC11 can promote tumor cell proliferation through various pathways. For example, in pituitary tumors and liver cancer, inhibition of HDAC11 increases p53 expression, thereby inducing tumor cell apoptosis [31, 32]. Additionally, HDAC11 negatively regulates LKB1 to inhibit the AMPK signaling pathway, promoting glycolysis and maintaining tumor cell stemness [33]. In breast cancer, HDAC11 promotes tumor cell proliferation and apoptosis by transcriptionally repressing the tumor suppressor gene ARHI [12]. Consequently, HDAC11 inhibitors have been proposed for cancer therapy. For instance, HDAC11 inhibitors suppress SOX2 expression, thereby reducing stemness in lung adenocarcinoma cells and overcoming drug resistance [34]. However, the role and molecular mechanisms of HDAC11 in tumor metastasis remain controversial. Patrick L. Leslie et al. reported that blocking HDAC11 in a breast cancer mouse model significantly increased distant metastasis of breast cancer cells, raising concerns about the use of HDAC11 inhibitors [28]. Nonetheless, the molecular mechanisms by which HDAC11 inhibits breast cancer metastasis have not been thoroughly investigated. Our study reveals that HDAC11 suppresses liver cancer cell metastasis by binding to the NuRD (MTA3) complex and inhibiting *TGFB1* expression. By encapsulating HDAC11 inhibitors and TGF-β1 inhibitors in lipid nanoparticle formulations, we can simultaneously suppress both the proliferation and metastasis of liver cancer cells, providing new strategies and methods for precision treatment of liver cancer.

## Conclusions

In this study, we found that HDAC11 promotes liver cancer cell proliferation, but its inhibition leads to an increase in liver cancer cell metastasis. To uncover the molecular mechanism underlying this phenotype, we employed immunoaffinity purification, silver staining, and mass spectrometry, revealing that HDAC11 specifically binds to the NuRD (MTA3) complex in vivo. This complex transcriptionally represses the expression of *TGFB1*, thereby inhibiting liver cancer cell metastasis. During the process of targeted HDAC11-based precision therapy for liver cancer, we utilized liposomal nanoparticles to co-encapsulate both the HDAC11 inhibitor and the TGF-β1 inhibitor. This formulation effectively suppresses liver cancer proliferation while also inhibiting metastasis. In summary, our study elucidates the molecular mechanism by which HDAC11 inhibits tumor metastasis (Fig. 7J). Furthermore, combined targeting of HDAC11 and TGFB1 significantly enhances the therapeutic efficacy against liver cancer, providing a novel approach for precision treatment of liver cancer.

## Materials and methods

### Cell culture and SiRNA transfection

The HCCLM3 cell line was obtained from the China Center for Type Culture Collection (Wuhan, China). MHCC97H cell lines were purchased from the Chinese Academy of Medical Sciences (Shanghai, China). HCCLM3 and MHCC97H cells were cultured in DMEM (Gibco, Cat. 11965092) supplemented with 10% fetal bovine serum (FBS, Gibco, Cat. C0232). All media were supplemented with 1% penicillin-streptomycin (Gibco, Cat. 15140122). Cells were maintained in a 37 °C incubator with 5% CO_2_. Lipofectamine RNAiMAX reagent (Invitrogen, USA, Cat. 13778150) and siRNA were mixed in Opti-MEM medium and incubated at room temperature for 10 min before being transfected into the suspended tumor cells. Cells were harvested for subsequent experiments 48 h post-transfection, and all experiments were repeated at least three times. siRNA sequences are listed in Supplementary Table S1.

### Antibodies and reagents

Histone Deacetylase 11 (HDAC11) antibody was purchased from Santa Cruz (sc-390737). b-Actin Mouse mAb (AC004), HRP-conjugated Goat anti-Mouse IgG (H + L) (AS003), and HRP-conjugated Goat anti-Rabbit IgG (H + L) (AS014) were obtained from ABclonal. Anti-FLAG^®^ M2 magnetic beads (M8823) were from Millipore. CoraLite594-conjugated Goat Anti-Mouse IgG (H + L) (SA00013-3), CoraLite594-conjugated Goat Anti-Rabbit IgG (H + L) (SA00013-4), CHD4 Polyclonal antibody (14173-1-AP), MTA3 Polyclonal antibody (14682-1-AP), and RbAp46 Polyclonal antibody (20365-1-AP) were purchased from Wuhan SanYing. Mouse monoclonal antibody E-cadherin (610181), Mouse monoclonal antibody α-catenin (610193), Mouse monoclonal antibody γ-catenin (610253), and Mouse monoclonal antibody N-cadherin (610920) were from BD Biosciences. Mouse monoclonal anti-Vimentin (V6630), Rabbit polyclonal anti-Fibronectin (F3648), and HDAC11 (H4539) were obtained from Sigma Aldrich. Recombinant Anti-TGFBI antibody (ab170874) was from Abcam. Pierce™ Protein A/G Agarose Magnetic Beads (78609) were from Thermo Fisher Scientific.

### Lentiviral infection

Recombinant lentiviruses carrying shHDAC11 and shSCR were constructed by GenePharma (Shanghai, China). After mixing the virus with Polybrene, the mixture was added to adherent cells, and after 48 h, the cells were collected. Stable cell lines were selected by continuing culture in media containing Puromycin. shRNA sequences are listed in Supplementary Table S2.

### EdU assay

Cells from the shHDAC11 and shSCR groups were seeded in 6-well plates at a density of 1 × 10^5 cells/ml and cultured overnight to allow adherence. The following steps were performed according to the BeyoClick™ EdU-555 Cell Proliferation Detection Kit (Beyotime, China, Cat. C0075S) instructions: EdU labeling was carried out, followed by fixation, washing, permeabilization, additional washing, DAPI staining, and finally, washing again before imaging the cells using a fluorescence microscope.

### Growth curve

Cells from the shHDAC11 and shSCR groups were seeded in 24-well plates at a density of 2000 cells/well. At specific time points, cells were trypsinized, counted using a cell counter, and the data was statistically analyzed.

### Colony formation assay

Cells were seeded in 6-well plates at a density of 1000 cells/well and the medium was changed every 2 days. For drug treatment, the drugs were added every 2 days. After 10–14 days of culture, the colonies were fixed with formaldehyde, stained with crystal violet, and photographed.

### Western blotting (WB)

Proteins were extracted from the treated cells using lysis buffer and denatured. The samples were then subjected to electrophoresis, electrotransfer, blocking, incubation with primary antibody, secondary antibody, and developed using a chemiluminescence reagent in a darkroom.

### Transwell assay

Transwell plates (Corning, Cat. 3422) coated with Matrigel (ABW, Cat. 082704) were used for invasion assays. Cells were transfected or pre-treated with drugs for 48 h, then resuspended in serum-free medium and seeded into the upper chamber at a density of 5 × 10^4 cells/300 µL. The lower chamber contained medium supplemented with FBS. After 18–24 h of incubation at 37 °C, cells on the upper side of the membrane were removed with a cotton swab. Cells on the lower side of the membrane were stained with crystal violet and counted.

### Wound healing assay

After treatment, cells were seeded in 6-well plates and allowed to adhere overnight. Once the cell density reached 90–100%, a scratch was made using a 200 µL yellow pipette tip, and the cells were cultured in serum-free medium. Images were captured at specific time points under a microscope.

### Immunopurification and mass spectrometry

FLAG-Vector and FLAG-HDAC11 plasmids were transfected into LM3 cells using TurboFect (Thermo Fisher Scientific, Cat. R0531). After 48 h, cell lysates expressing FLAG-Vector and FLAG-SMYD3 were incubated with ANTI-FLAG^®^ M2 magnetic beads at room temperature for 1 h. The beads were washed, and proteins were eluted using Flag Peptide (Sigma-Aldrich, F4799). The eluted proteins were separated by SDS-PAGE, silver-stained using the Thermo Scientific Pierce Silver Stain Kit (24612), and analyzed by LC-MS/MS.

### Immunoprecipitation (IP)

Cell lysates were incubated overnight with primary antibody at 4 °C. The samples were then mixed with Pierce™ Protein A/G Agarose Magnetic Beads (Thermo Fisher Scientific, 78609) at 4 °C for 2 h. After five washes, the bead-protein complexes were separated by SDS-PAGE and analyzed by WB.

### Immunofluorescence

EGFP-HDAC11 (NM_024827.4) plasmid was synthesized by Hun an Fenghui Biotechnology (Hunan, China). Cells were seeded in 6-well plates and transfected with EGFP-HDAC11 plasmid within 24 h. After 48 h, cells were fixed, permeabilized, blocked with 1% BSA, incubated with primary antibody, then with fluorescent secondary antibody, and stained with DAPI for nuclei. Images were captured using a Leica laser scanning confocal microscope.

#### Glutathione S‑transferase (GST) pull‑down experiments

GST fusion proteins were expressed in BL21 Escherichia coli and bacterial cells were lysed by sonication in ice-cold PBS supplemented with a protease inhibitor cocktail. In vitro transcription and translation were performed using rabbit reticulocyte lysate (Promega, USA, Cat. No. L1170). For the GST pull-down assays, approximately 10 µg of the appropriate GST fusion proteins were incubated with 5–8 µL of the in vitro-translated products in binding buffer (PBS containing 0.8% BSA and a protease inhibitor cocktail). The binding reactions were then added to 30 µL of glutathione‑Sepharose beads and incubated at 4 °C for 2 h with gentle rotation. After incubation, the beads were washed five times with binding buffer, resuspended in 30 µL of 2 × SDS–PAGE loading buffer, and resolved on 10% SDS–PAGE gels. Protein levels were subsequently detected by Western blotting using specific antibodies.

### RT-qPCR

Total RNA was extracted using the RNA-Quick Purification Kit (ES Science, Cat. RN001) according to the manufacturer’s instructions. cDNA was synthesized using the Reverse Transcription Kit (Roche, Cat. 04897030001). Real-time PCR was performed using SYBR Green fluorescence on the 7500 Detection System. GAPDH was used as the internal control. The primers used are listed in Supplementary Table S3.

#### RNA-seq data analysis

siRNA-transfected liver cancer cells were harvested 48 h post-transfection, and RNA was extracted for quality control and sequencing by the BGIseq500 platform (BGI-Shenzhen, China). Differential expression analysis was performed using DESeq2 with a threshold of |log2FC| > 1 (FC: fold change) and *p* ≤ 0.05. Heatmaps were generated using R package pheatmap. KEGG and GO analyses were conducted using the DAVID Database (https://davidbioinformatics.nih.gov/), and GSEA was performed using R package GSEABase.

#### Tumor xenografts

Stable LM3 cells expressing shHDAC11 or shSCR were subcutaneously injected into immunodeficient mice at a dose of 1 × 10^6 cells/100 µL. Five animals per group were used for each experiment. Tumor volume was measured every 2 days using calipers and calculated using the formula: (length×width²) / 2. All animal experiments were approved by the Lishui College Animal Ethics Committee (Approval No. 2024YD0073).

#### In vivo metastasis in NOD/SCID mice

Stable LM3 cells expressing firefly luciferase from the control shRNA or shHDAC11 group were injected into the tail vein of NOD/SCID mice. Lung metastasis was monitored weekly using an in vivo bioluminescence imaging system.

### ChIP and Re-ChIP

ChIP was performed according to the kit’s instructions. Briefly, cells were crosslinked with 1% formaldehyde, and chromatin was sheared and subjected to chromatin immunoprecipitation, followed by elution, de-crosslinking, and DNA purification. For Re-ChIP, ChIP complexes were eluted with DTT at 37 °C for 30 min, and the eluted material was diluted 30 times for a second round of ChIP. The primers used are listed in Supplementary Table S4.

### Preparation of liposomal nanoparticles

E80 (4.92 mg), Chol (1.25 mg), 0.75 mg iRGD, 0.60 mg TGF-β1 inhibitor, and 0.60 mg HDAC11 inhibitor were dissolved in 4 mL of a chloroform: methanol mixture (3:1). The resulting mixture was placed in a 50 mL round-bottom flask, and after thorough mixing, the solvent was evaporated by rotary evaporation at 45 °C for 45 min to remove the organic solvents, resulting in a lipid film. The lipid film was then hydrated with PBS at room temperature. The hydrated lipid solution was transferred into a 10 mL vial, which was subsequently placed in a 50 mL beaker filled with ice. Ultrasonication was performed using a cell disruptor with a probe (300 W, 2 s on, 3 s off, 8 min total), yielding a liposomal solution containing the desired compounds. In the mouse subcutaneous tumor xenograft and lung metastasis models, animals were treated with Lip (Ele + EW) nanoparticles at a dose of 80 mg/kg, with liposomes at the same dose (80 mg/kg) serving as the control.

## Characterization of liposomal nanoparticles

1 mL of diluted liposomal solution was transferred into a size measurement tube, and the particle size of the liposomal nanoparticles was measured using a nanoparticle size and zeta potential analyzer (Zetasizer Lab). After measuring the particle size, the solution was transferred into a zeta potential measurement tube to assess the zeta potential of the liposomal nanoparticles. The encapsulation efficiency (EE) and drug loading (DL) of the liposomal nanoparticles were calculated by measuring the absorbance using a UV-Vis spectrophotometer (Shimadzu, UV1900i). The morphology of the liposomal nanoparticles was observed using transmission electron microscopy (TEM).

### Statistical analysis

Unless otherwise specified, data are expressed as Mean ± SD. Statistical comparisons were made using an unpaired t-test. Statistical significance was indicated as follows: *p < 0.05; **p < 0.01; ***p < 0.001, as indicated in the figures.

## Supplementary Information

Below is the link to the electronic supplementary material.

Supplementary Material 1

Supplementary Material 2: Supplementary figure 1. Hematoxylin and eosin (HE) staining of lung tissues from mice to detect metastatic foci in different groups. B. KEGG pathway analysis of the HDAC11 interacting proteins identified by mass spectrometry. C. Immunoprecipitation analysis of HDAC11 with MTA1 and MTA2. Immunoprecipitation assays were performed in MHCC97H and HCCLM3 cells using antibodies against MTA1 or MTA2. The immunoprecipitates were subsequently analyzed by western blotting with an anti-HDAC11 antibody to detect the interactions between HDAC11 and MTA1 or MTA2 proteins.

Supplementary Material 3

## Data Availability

The supporting data and materials are provided in Additional Tables. RNA-seq data had been deposited in CNCB (https://ngdc.cncb.ac.cn/gsub/) with the accession number PRJCA036093.
