# Supplementary material for: HDAC11 interacts with the NuRD (MTA3) complex to transcriptionally suppress TGFβ1 expression and inhibit hepatocellular carcinoma metastasis
Source: Clin Epigenetics. 2026 Jan 17;18:29. doi: 10.1186/s13148-026-02050-y (PMC12895668; doi:10.1186/s13148-026-02050-y)
Supplement: Supplementary file 1 — Supplementary Material 1 [file 13148_2026_2050_MOESM1_ESM.docx]

Supplementary Table S1. siRNA sequences

| Control siRNA | TTCTCCGAACGTGTCACGT |
| --- | --- |
| HDAC11-siRNA 1 | CAGTTAACTGAGAATTGGA |
| HDAC11-siRNA 2 | GGCGCTATCTTAATGAGCT |
| MTA3-siRNA 1 | GGAAGAATCTGAAACAACA |
| MTA3-siRNA 2 | GTGGCAACGTGGAAGCAAA |
| TGFβ1-siRNA | CCAGAAAUACAGCAACAAUTT |

Supplementary Table S2. shRNA sequences

| HDAC11-shRNA Control | TTCTCCGAACGTGTCACGT |
| --- | --- |
| HDAC11-shRNA 1 | GCACACGAGGCGCTATCTTAA |
| HDAC11-shRNA 2 | GGATGATGAGTACCTGGATAA |

Supplementary Table S3: Real-time quantitative primers used in this study

| gene | strand | sequence |
| --- | --- | --- |
| *HDCA11*-RT | F | CCAGACAGGAGGAACCATAA |
| *HDAC11*-RT | R | TCCGCATAGGCACAGAA |
| *GAPDH*-RT | F | GGAGCGAGATCCCTCCAAAAT |
| *GAPDH*-RT | R | GGCTGTTGTCATACTTCTCATGG |
| *TGFβ1*-RT | F | AACTGAAAGAGGCTGAGACC |
| *TGFβ1*-RT | R | AAGTTCGTTTAGTGTCTGATCC |
| *BDNF*-RT | F | GGCTTGACATCATTGGCTGAC |
| *BDNF-*RT | R | CATTGGGCCGAACTTTCTGGT |
| C*CN1*-RT | F | GCGAGGAGTGGGTCTGTGAC |
| *CCN1*-RT | R | CTTGTAAAGGGTTGTATAGGATGC |
| *SERPINE1*-RT | F | ATCGAGGTGAACGAGAGTGG |
| S*ERPINE1*-RT | R | ACTGTTCCTGTGGGGTTGTG |
| *LAMC2*-RT | F | GATTGAACAGGAGATTGGGA |
| *LAMC2*-RT | R | TTCTGGGCTTCTGTAATCAC |
| *VIM*-RT | F | ATTGAGATTGCCACCTACAG |
| *VIM*-RT | R | ATCCAGATTAGTTTCCCTCAG |
| *BGN*-RT | F | ATCAGGATGATCGAGAACGG |
| *BGN*-RT | R | AGATAGACCACCTGGAGGAG |
| *AREG*-RT | F | GAGTGAAATGCCTTCTAGTAGTG |
| *AREG*-RT | R | TACCTGTTCAACTCTGACTG |
| *COL1A1-*RT | F | CTGGAAGAGTGGAGAGTACTG |
| *COL1A1-*RT | R | GTAGGTGATGTTCTGGGAGG |
| *LOXL2*-RT | F | ACCAGATAGAGAACCTGAATATCC |
| *LOXL2-*RT | R | CACTTTGGTATTGTATGTCCTCTC |
| *ATF3*-RT | F | CTGGAATCAGTCACTGTCAG |
| *ATF3*-RT | R | CTTCTCCGACTCTTTCTGCA |
| *TIMP3*-RT | F | AAGCAGATGAAGATGTACCGA |
| *TIMP3-*RT | R | GTGATACCGATAGTTCAGCC |
| *BMP2*-RT | F | CTACATGCTAGACCTGTATCG |
| *BMP2-*RT | R | CCAAAGATTCTTCATGGTGG |
| *CASP7-*RT | F | ACCCAAACTCTTCTTCATTCAG |
| *CASP7*-RT | R | AGTAATAGCCTGGAACCGTG |
| *MTA3-*RT | F | AAGGTGAAATCAGAGTGGGA |
| *MTA3-*RT | R | CCATAGCGTGAAACAAGGTG |

Supplementary Table S4. The Primers used in qChIP Assays

| gene | strand | sequence |
| --- | --- | --- |
| *TGF-β1-*ChIP | F | CCGCCTGGTCCTCTTTC |
| *TGF-β1-*ChIP | R | CCACCGTCCTCATCTCG |
| *AREG-*ChIP | F | TAAATGCCAGTATGCCACC |
| *AREG-*ChIP | R | AGCCGATAATGTTCAAAGC |
| *TIMP3*-ChIP | F | GGATGGCACTTCAGGG |
| *TIMP3*-ChIP | R | AACAAACACGGTTCAGGAT |
| *ATF3*-ChIP | F | ACTGGCAACACGGAGTAAA |
| *ATF3*-ChIP | R | AGCACCGAGGACTCACG |
| *CASP7*-ChIP | F | GAGCAGCATCTCCACCAGC |
| *CASP7*-ChIP | R | GGGACTCCACGCCTACATTA |
