# Supplementary figures and images for: HDAC11 interacts with the NuRD (MTA3) complex to transcriptionally suppress TGFβ1 expression and inhibit hepatocellular carcinoma metastasis
Source: Clin Epigenetics. 2026 Jan 17;18:29. doi: 10.1186/s13148-026-02050-y (PMC12895668; doi:10.1186/s13148-026-02050-y)

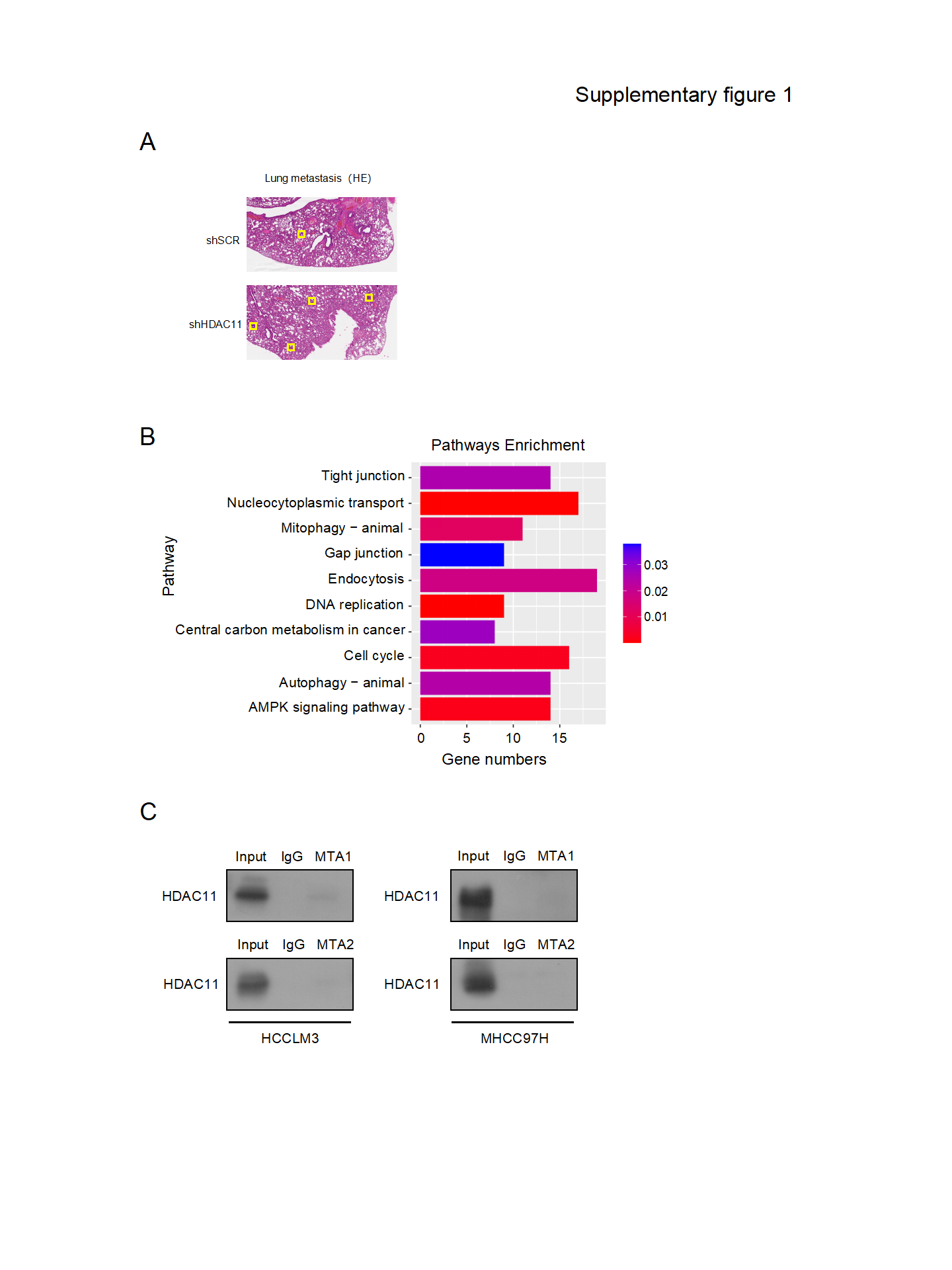

Supplement: Supplementary file 2 — Supplementary Material 2: Supplementary figure 1. Hematoxylin and eosin (HE) staining of lung tissues from mice to detect metastatic foci in different groups. B. KEGG pathway analysis of the HDAC11 interacting proteins identified by mass spectrometry. C. Immunoprecipitation analysis of HDAC11 with MTA1 and MTA2. Immunoprecipitation assays were performed in MHCC97H and HCCLM3 cells using antibodies against MTA1 or MTA2. The immunoprecipitates were subsequently analyzed by western blotting with an anti-HDAC11 antibody to detect the interactions between HDAC11 and MTA1 or MTA2 proteins. [file 13148_2026_2050_MOESM2_ESM.tif]
