# Supplementary material for: HDAC11 interacts with the NuRD (MTA3) complex to transcriptionally suppress TGFβ1 expression and inhibit hepatocellular carcinoma metastasis
Source: Clin Epigenetics. 2026 Jan 17;18:29. doi: 10.1186/s13148-026-02050-y (PMC12895668; doi:10.1186/s13148-026-02050-y)
Supplement: Supplementary file 3 — Supplementary Material 3 [file 13148_2026_2050_MOESM3_ESM.pptx]

## Slide 1
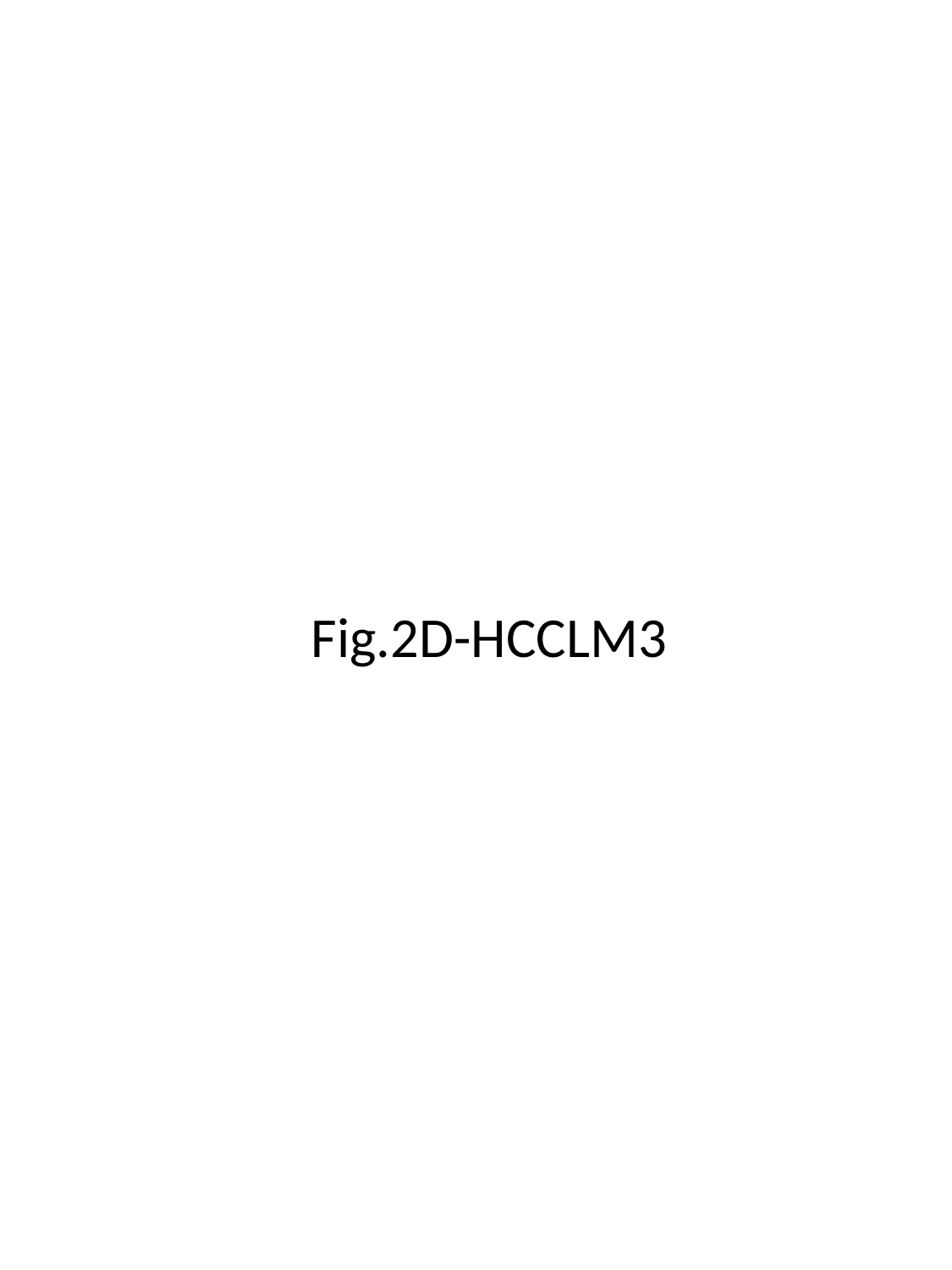

Fig.2D-HCCLM3

## Slide 2
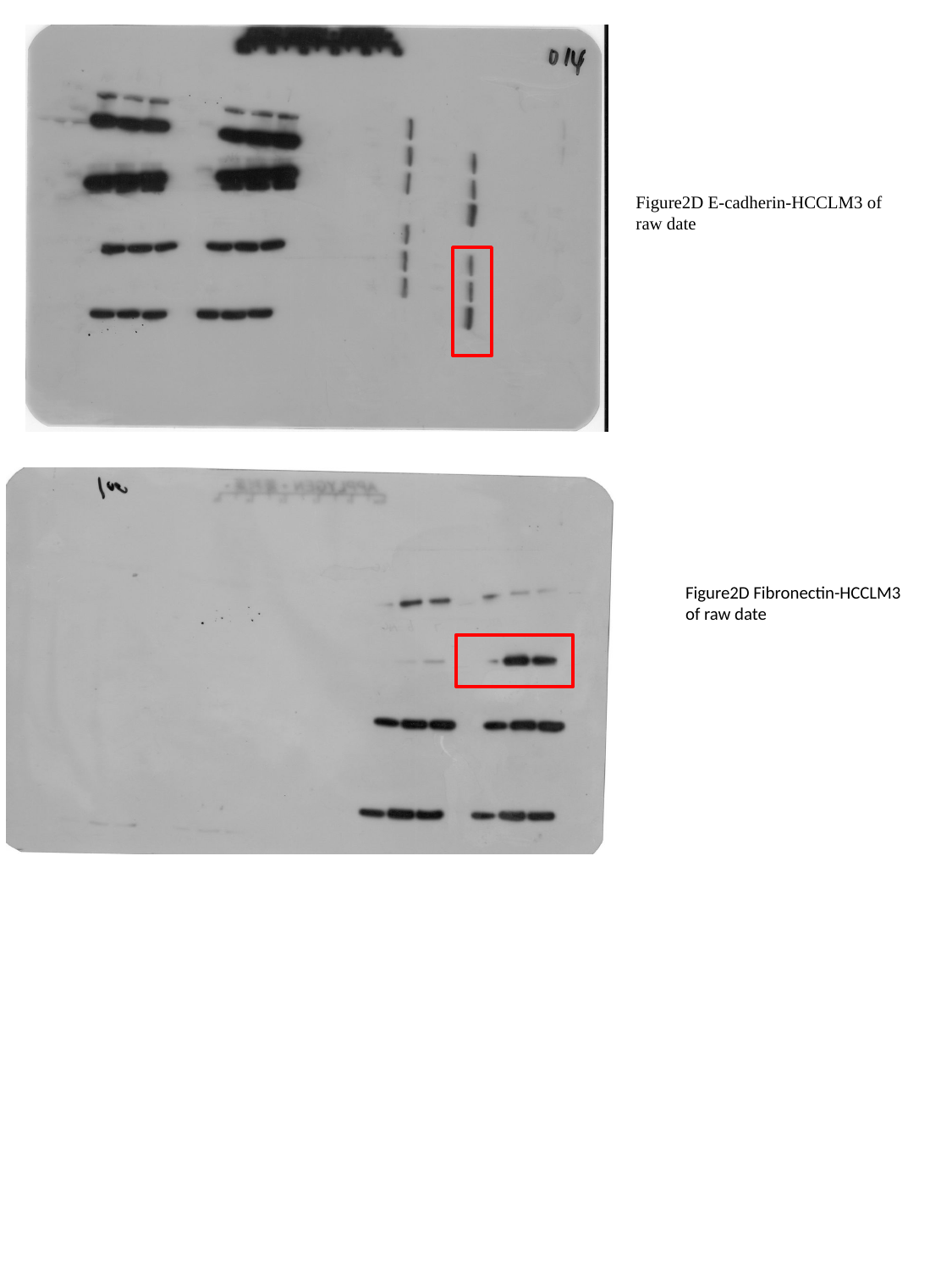

Figure2D E-cadherin-HCCLM3 of raw date
Figure2D Fibronectin-HCCLM3 of raw date

## Slide 3
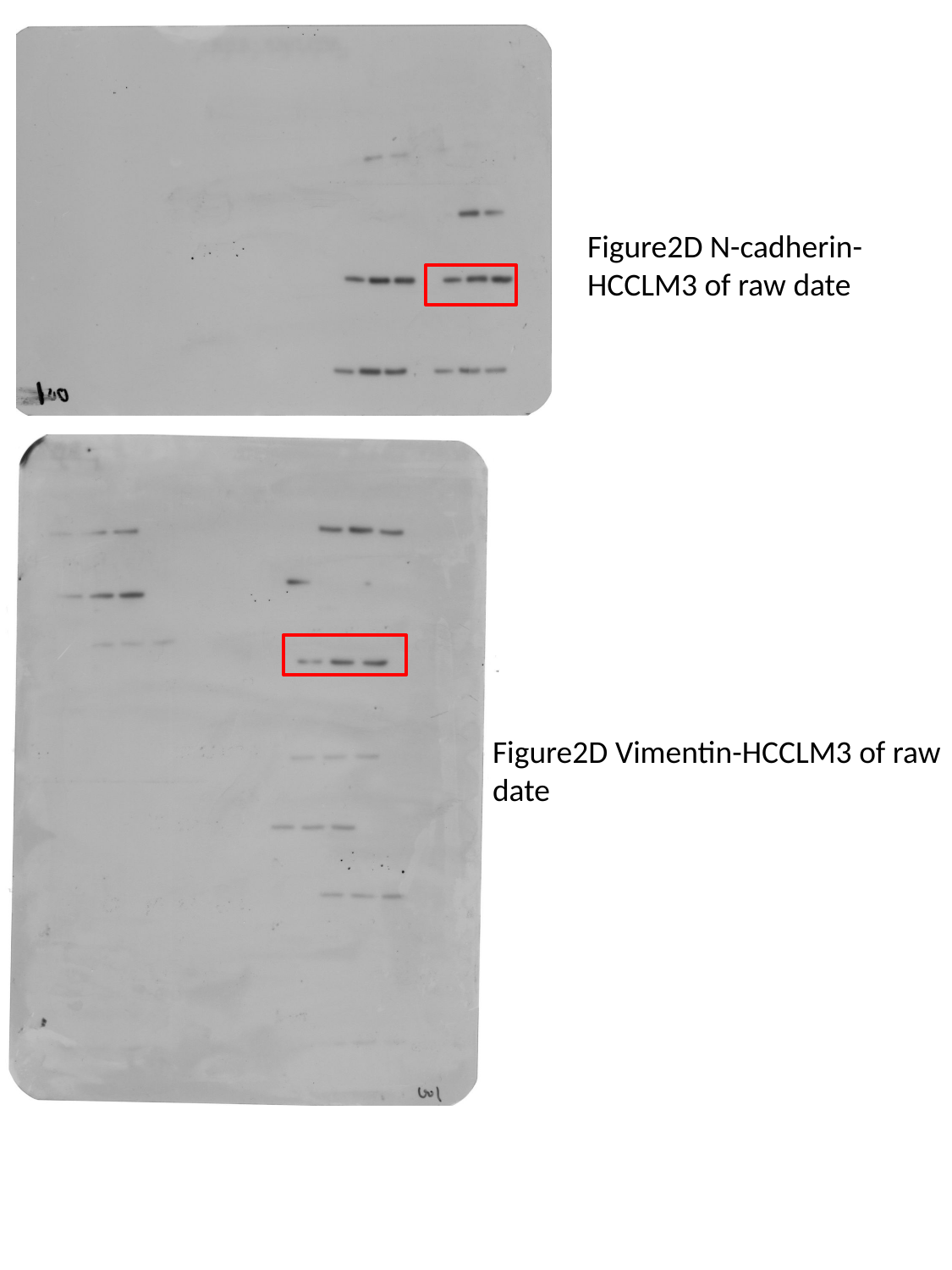

Figure2D N-cadherin-HCCLM3 of raw date
Figure2D Vimentin-HCCLM3 of raw date

## Slide 4
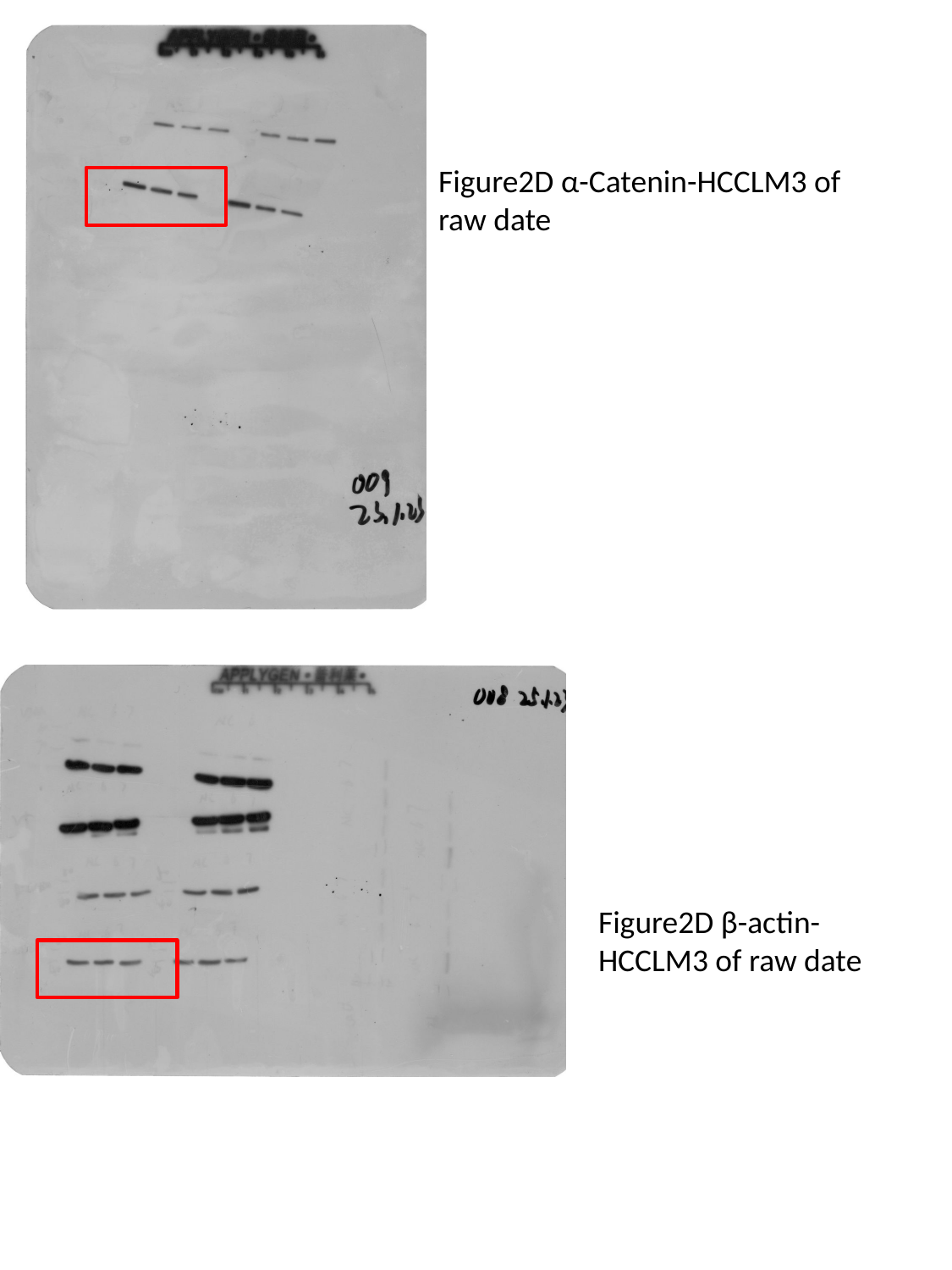

Figure2D α-Catenin-HCCLM3 of raw date
Figure2D β-actin-HCCLM3 of raw date

## Slide 5
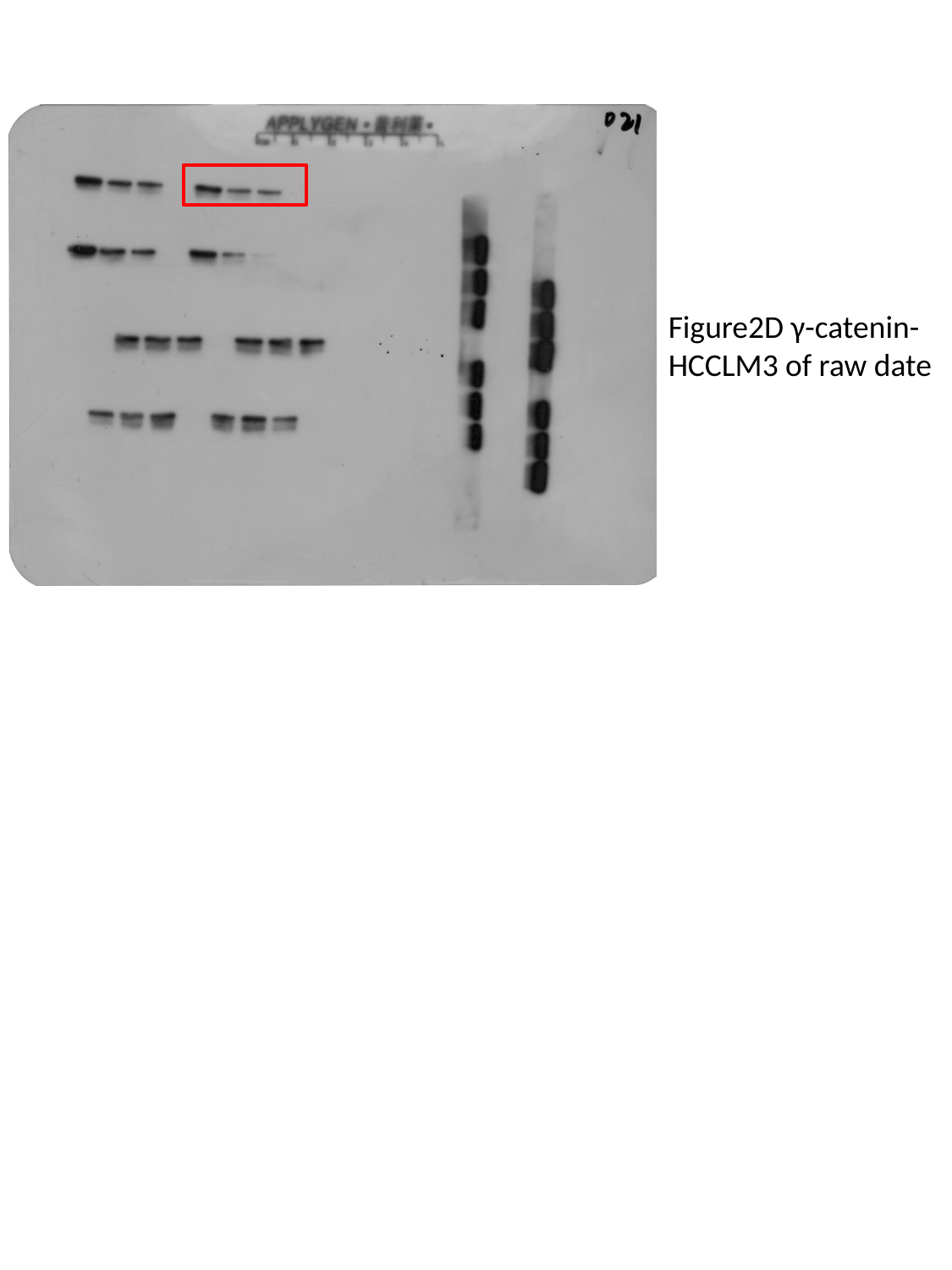

Figure2D γ-catenin-HCCLM3 of raw date

## Slide 6
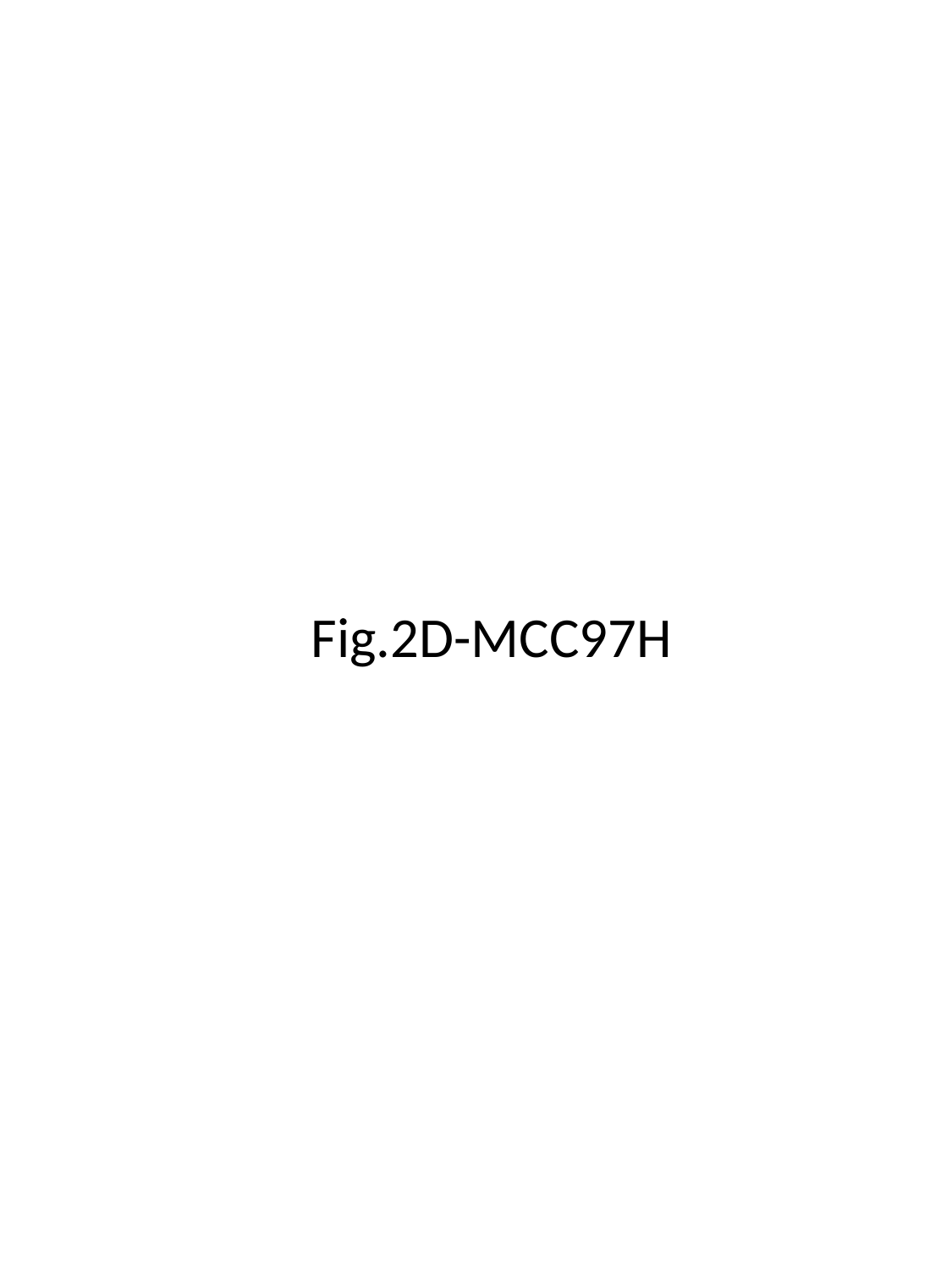

Fig.2D-MCC97H

## Slide 7
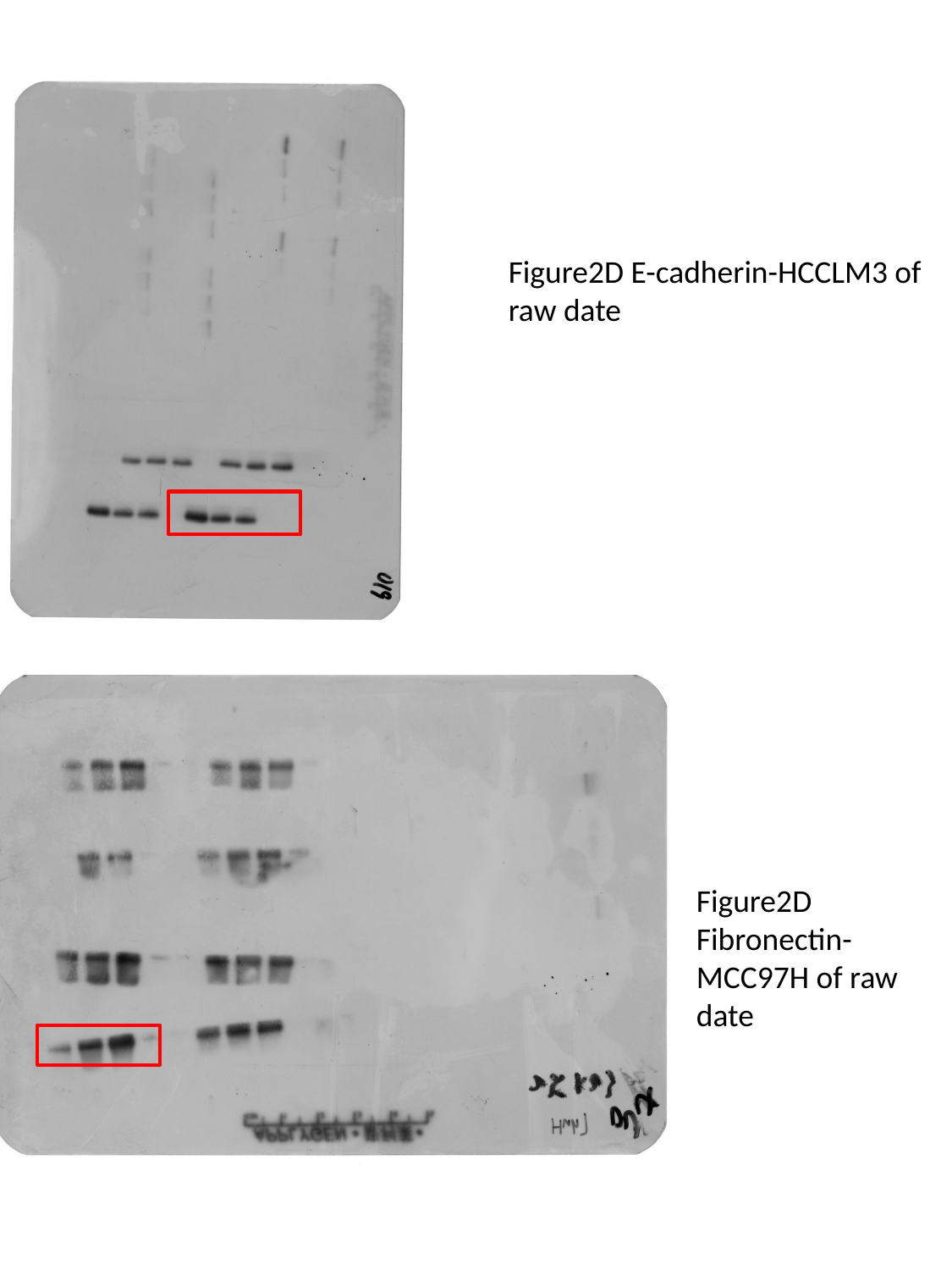

Figure2D E-cadherin-HCCLM3 of raw date
Figure2D Fibronectin-MCC97H of raw date

## Slide 8
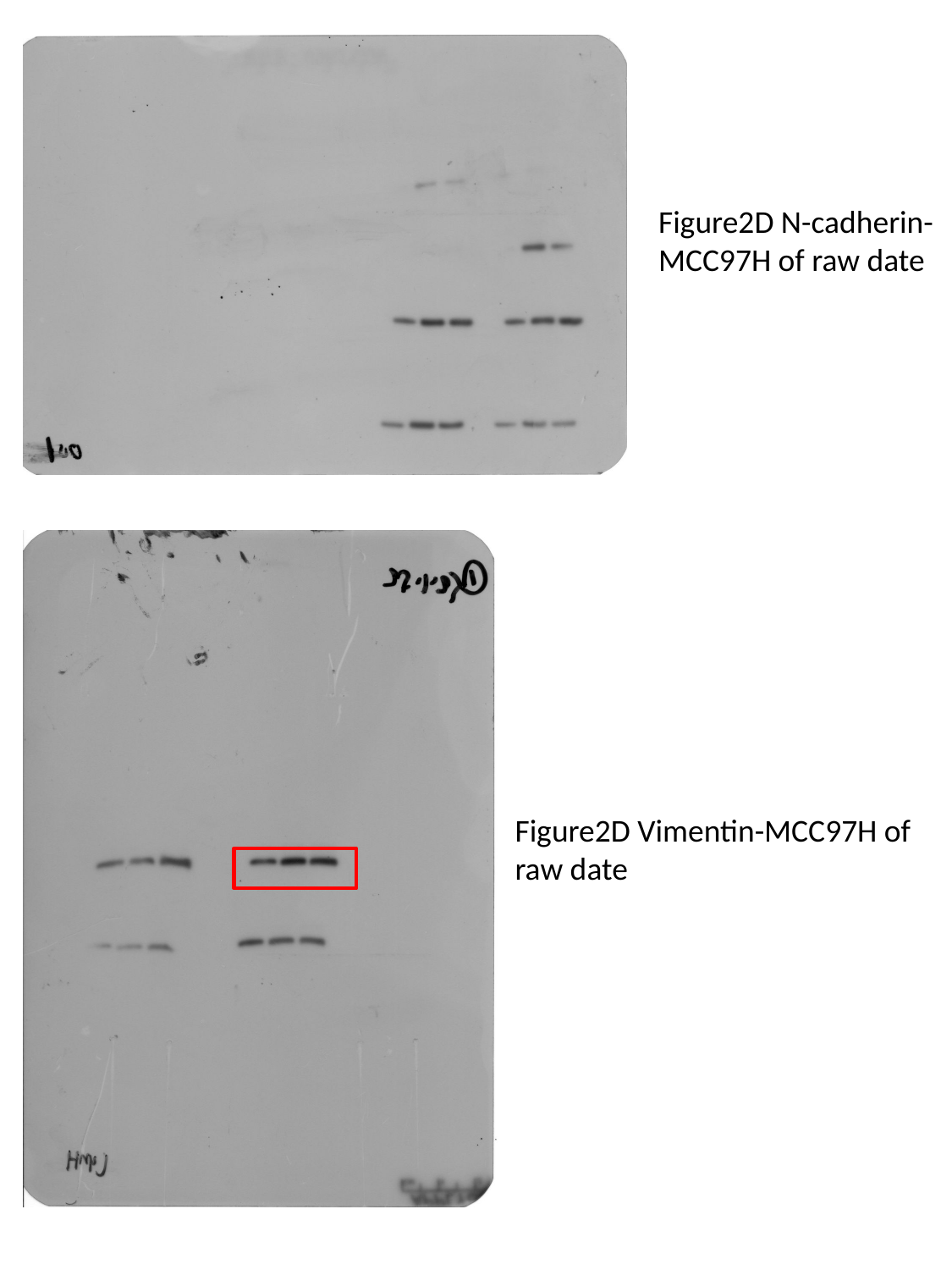

Figure2D N-cadherin-MCC97H of raw date
Figure2D Vimentin-MCC97H of raw date

## Slide 9
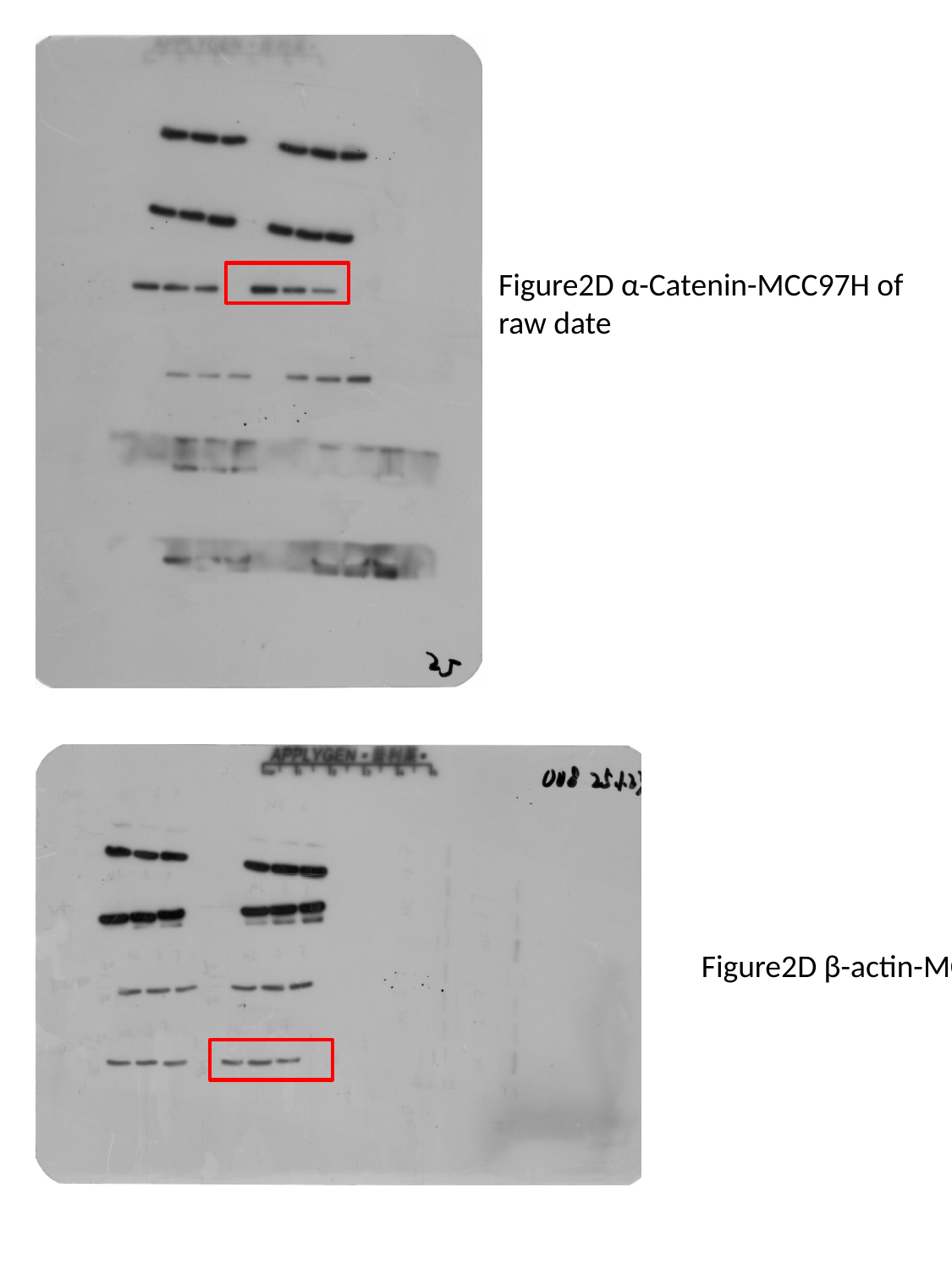

Figure2D α-Catenin-MCC97H of raw date
Figure2D β-actin-MCC97H of raw date

## Slide 10
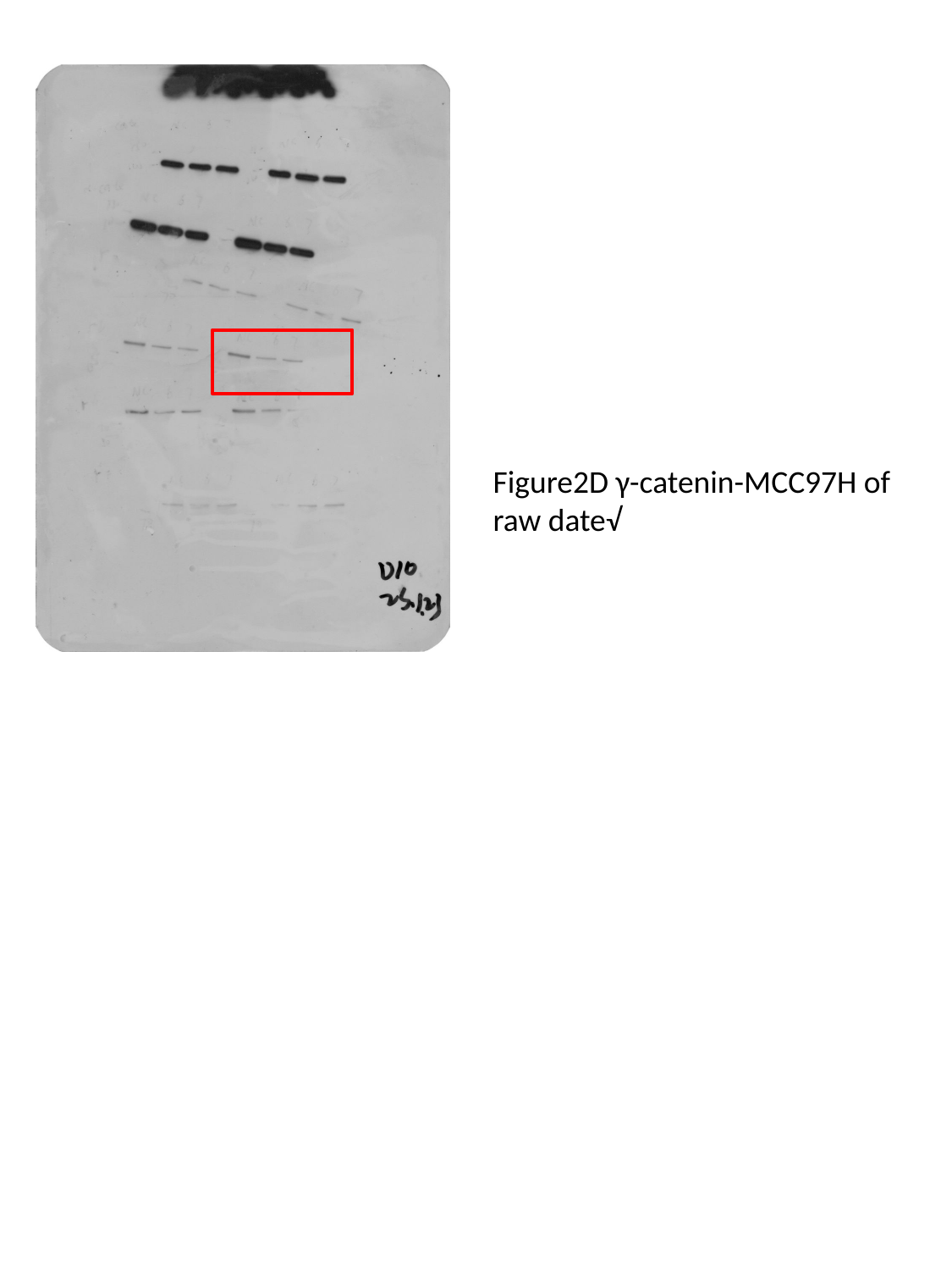

Figure2D γ-catenin-MCC97H of raw date√

## Slide 11
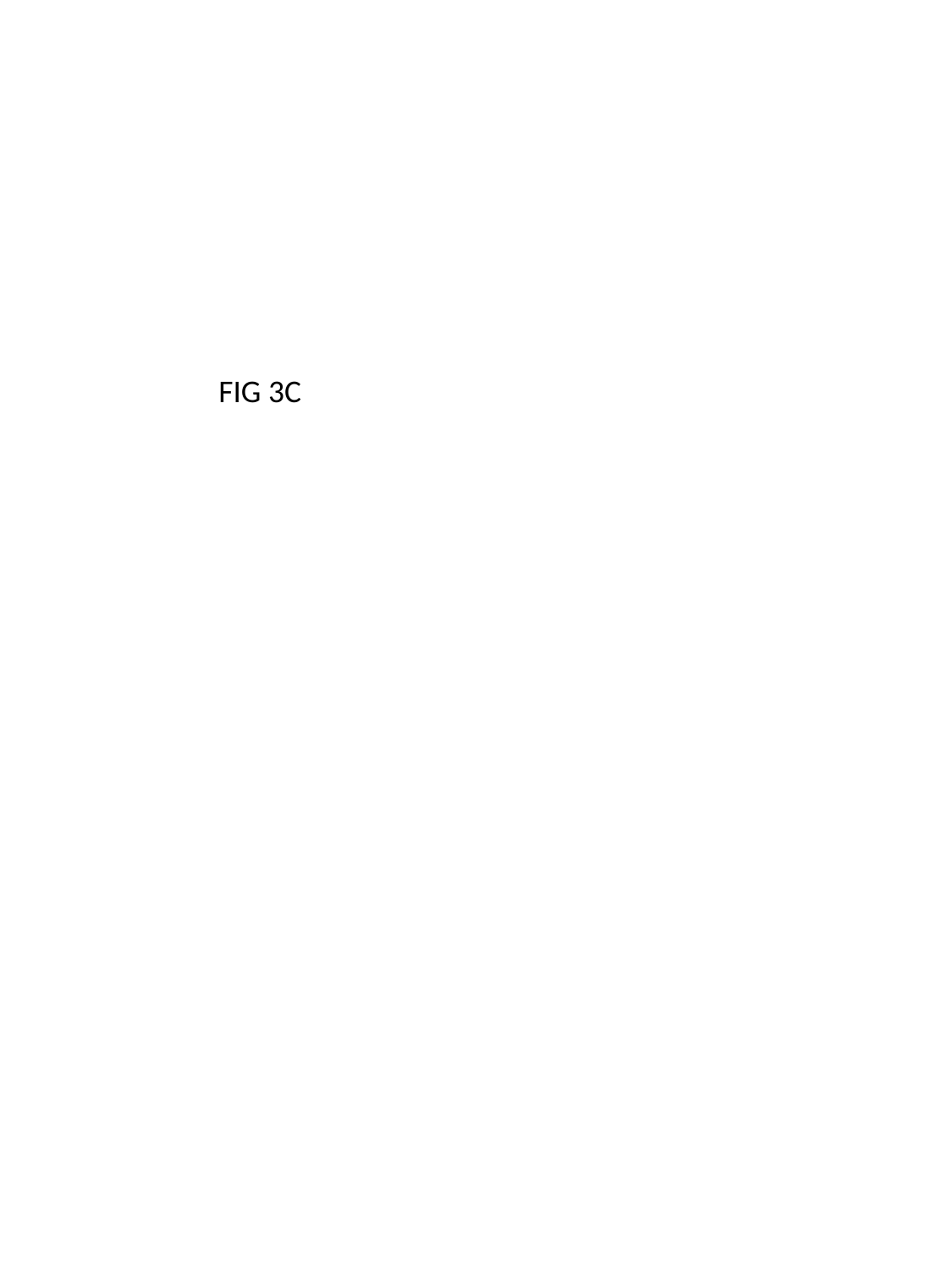

FIG 3C

## Slide 12
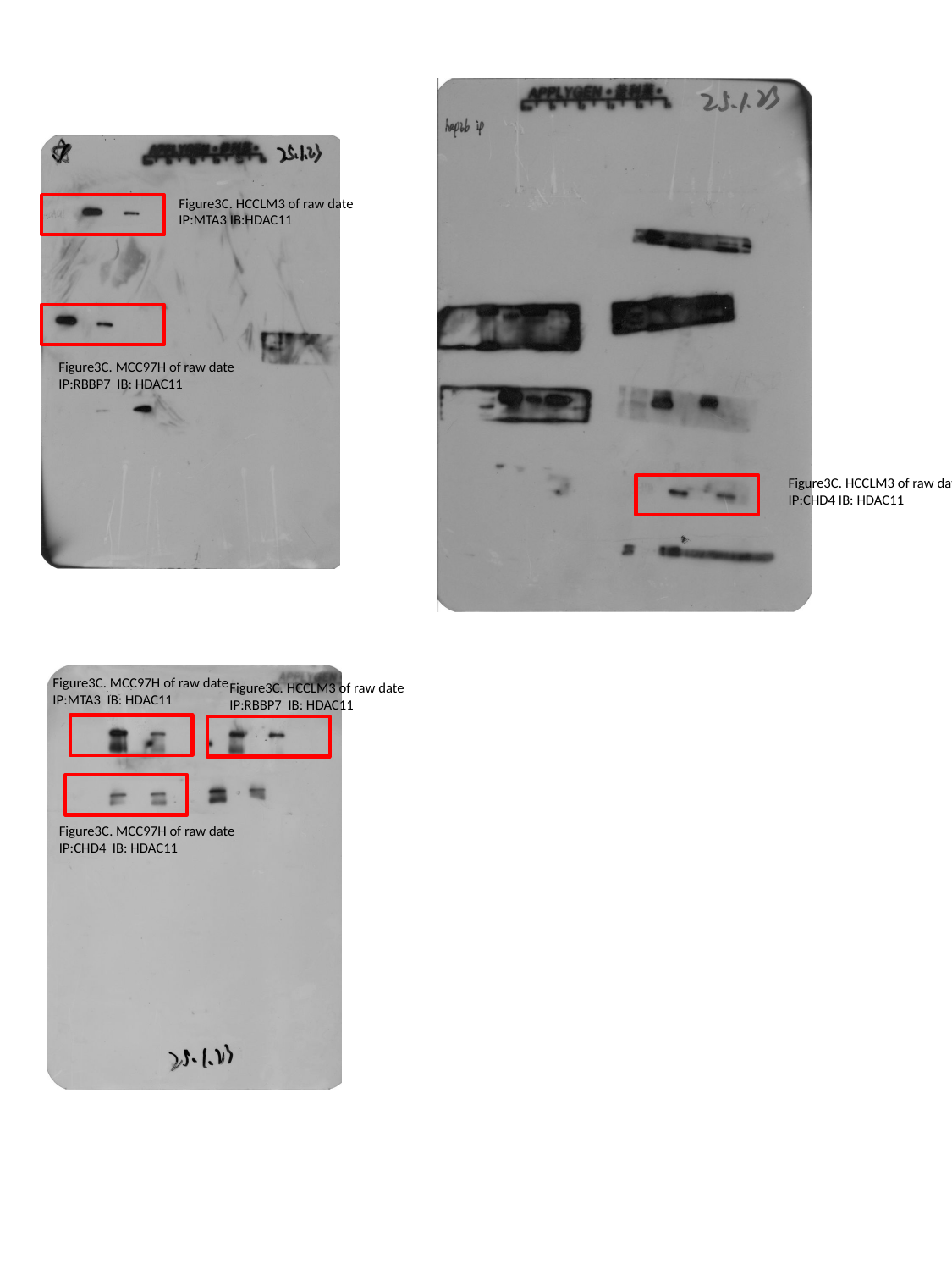

Figure3C. HCCLM3 of raw date
IP:MTA3 IB:HDAC11
Figure3C. MCC97H of raw date
IP:RBBP7 IB: HDAC11
Figure3C. HCCLM3 of raw date
IP:CHD4 IB: HDAC11
Figure3C. MCC97H of raw date
IP:MTA3 IB: HDAC11
Figure3C. HCCLM3 of raw date
IP:RBBP7 IB: HDAC11
Figure3C. MCC97H of raw date
IP:CHD4 IB: HDAC11

## Slide 13
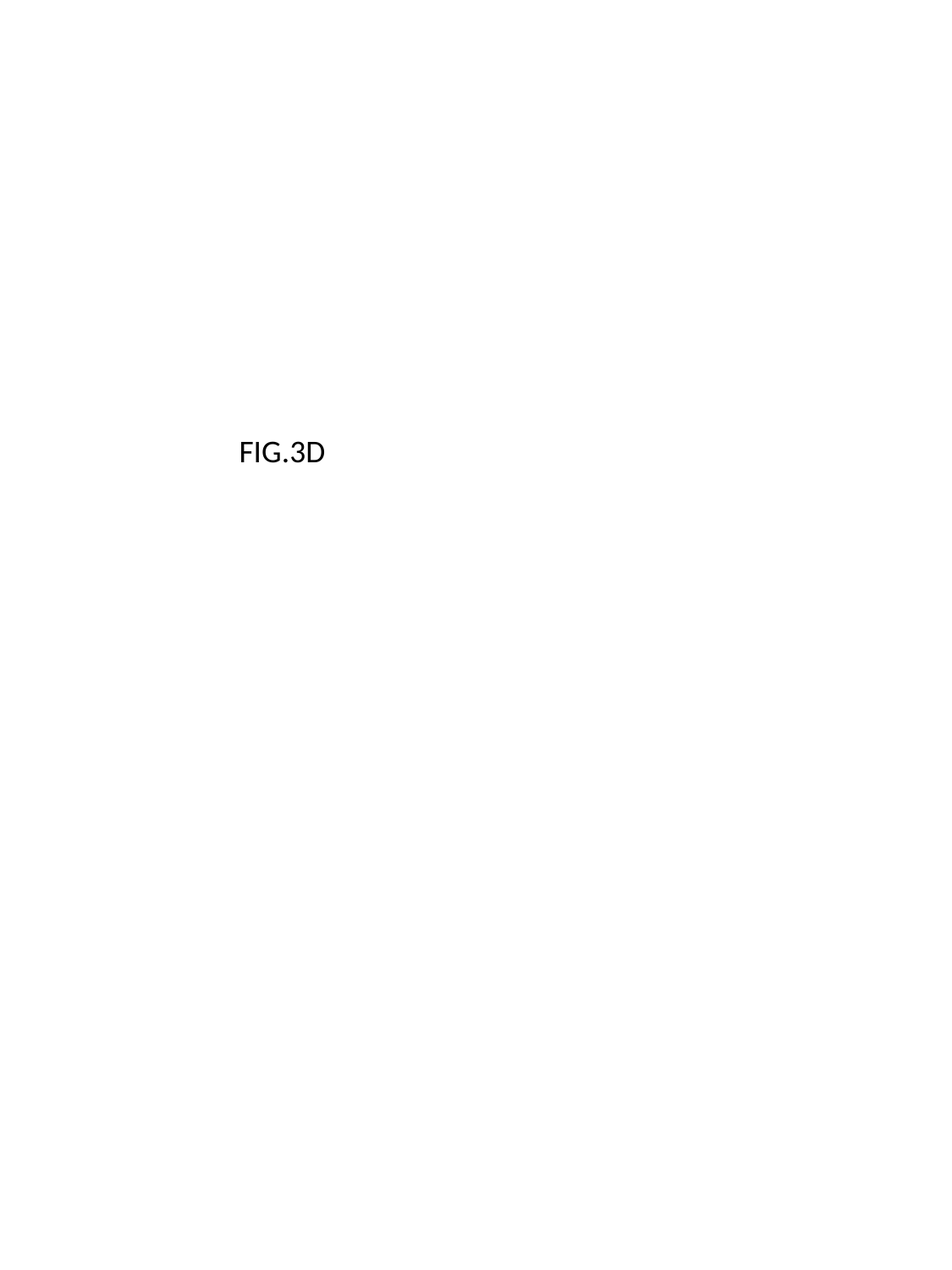

FIG.3D

## Slide 14
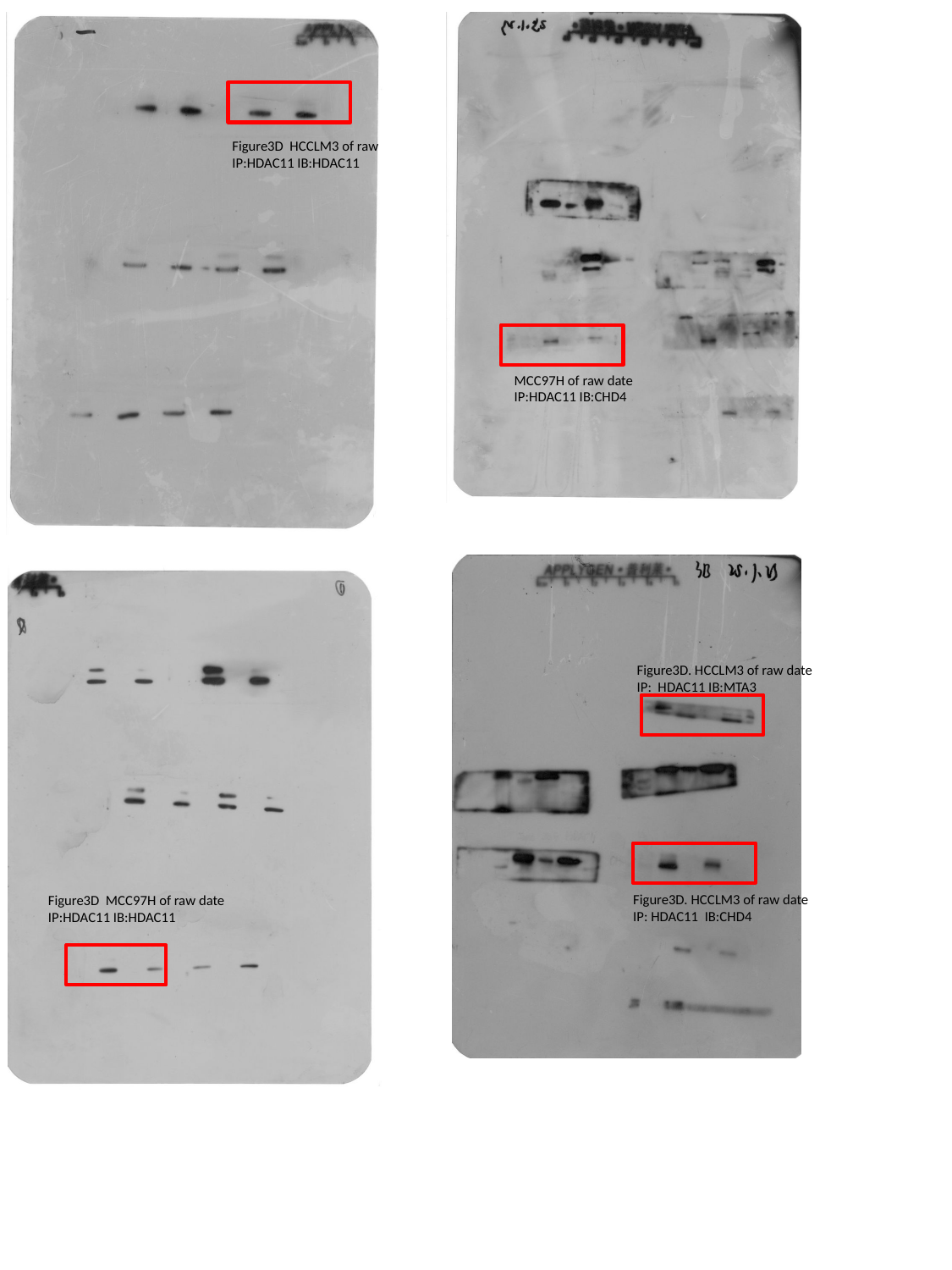

Figure3D HCCLM3 of raw
IP:HDAC11 IB:HDAC11
MCC97H of raw date
IP:HDAC11 IB:CHD4
Figure3D. HCCLM3 of raw date
IP: HDAC11 IB:MTA3
Figure3D. HCCLM3 of raw date
IP: HDAC11 IB:CHD4
Figure3D MCC97H of raw date
IP:HDAC11 IB:HDAC11

## Slide 15
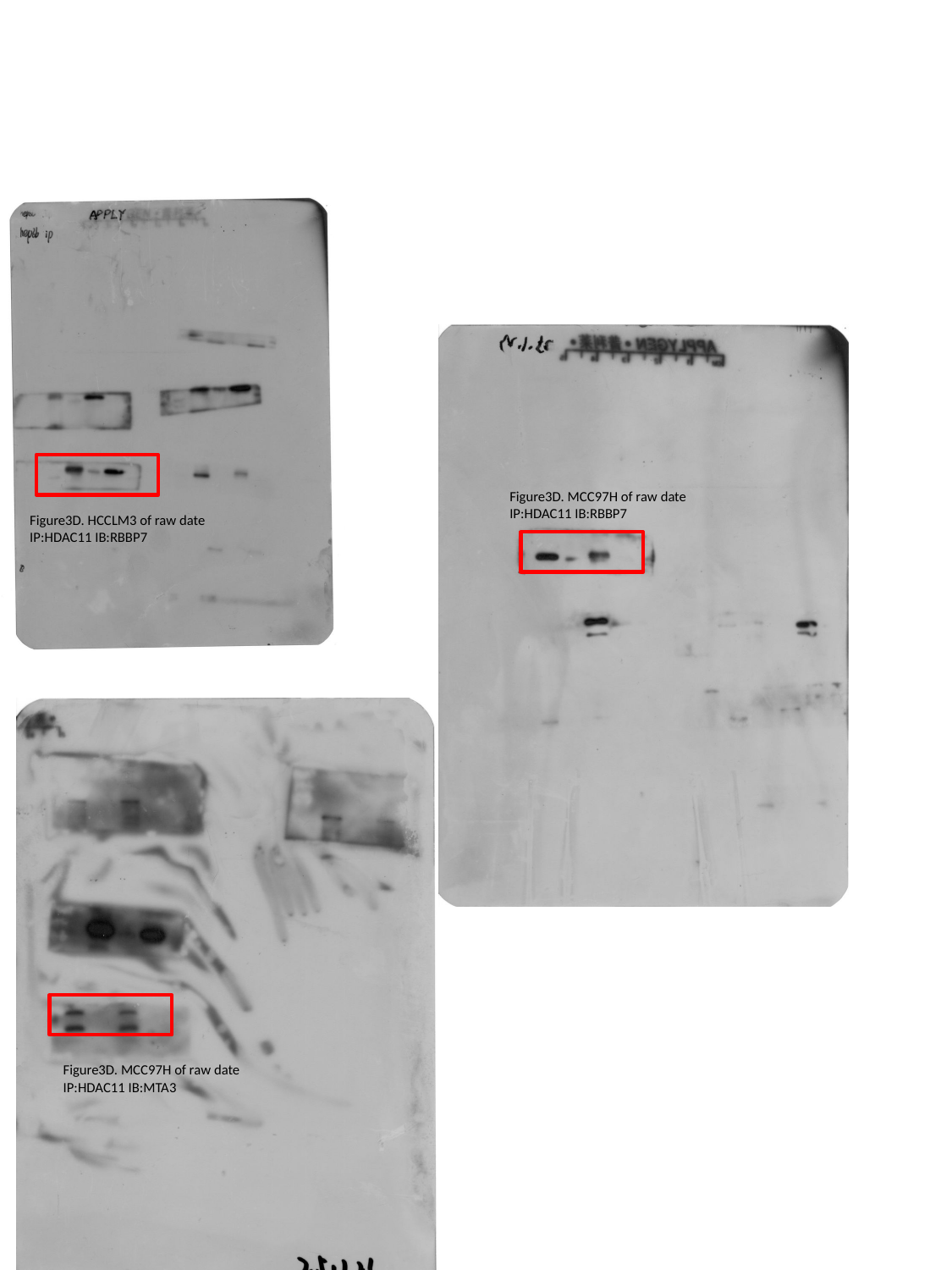

Figure3D. MCC97H of raw date
IP:HDAC11 IB:RBBP7
Figure3D. HCCLM3 of raw date
IP:HDAC11 IB:RBBP7
Figure3D. MCC97H of raw date
IP:HDAC11 IB:MTA3

## Slide 16
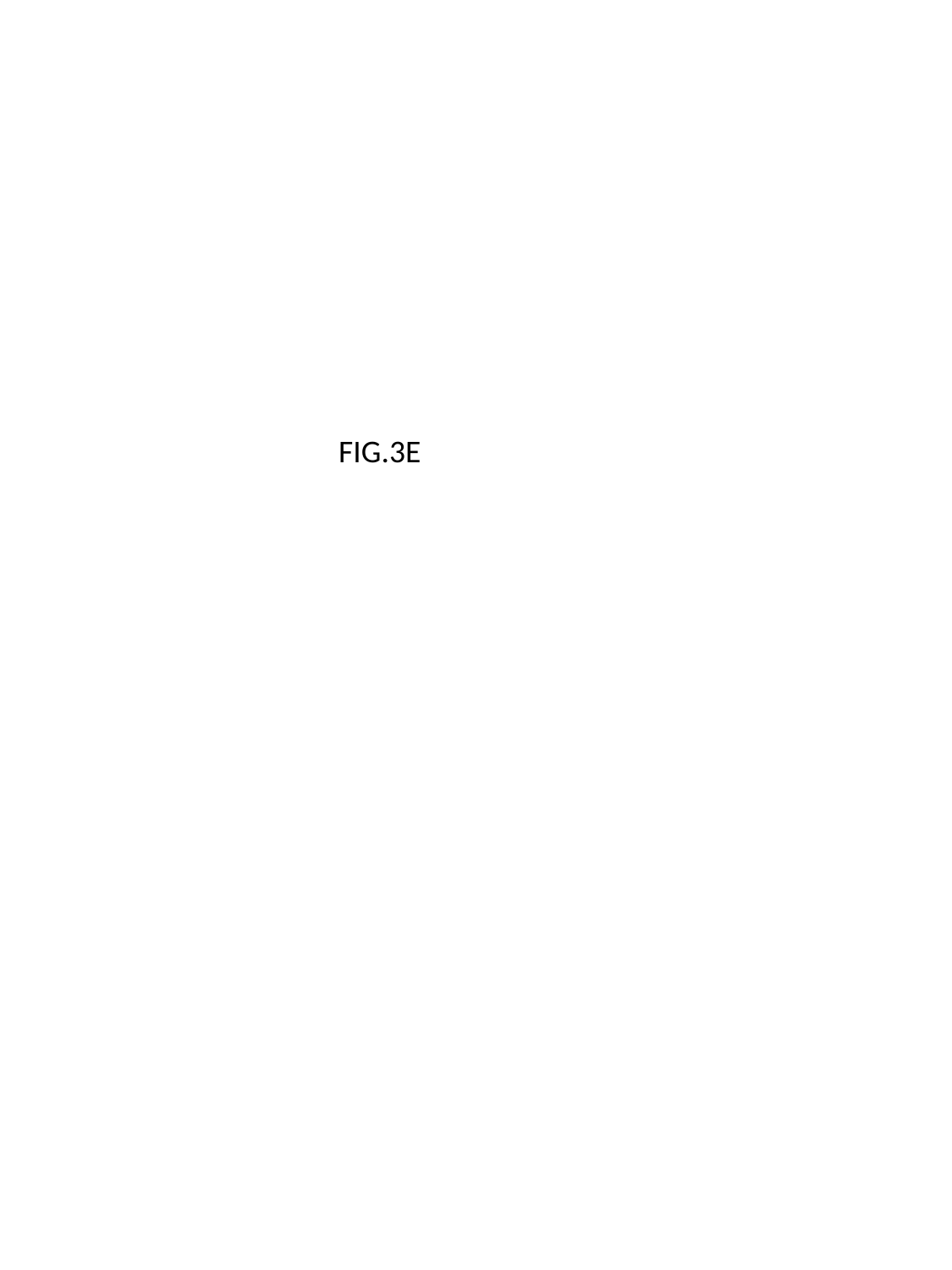

FIG.3E

## Slide 17
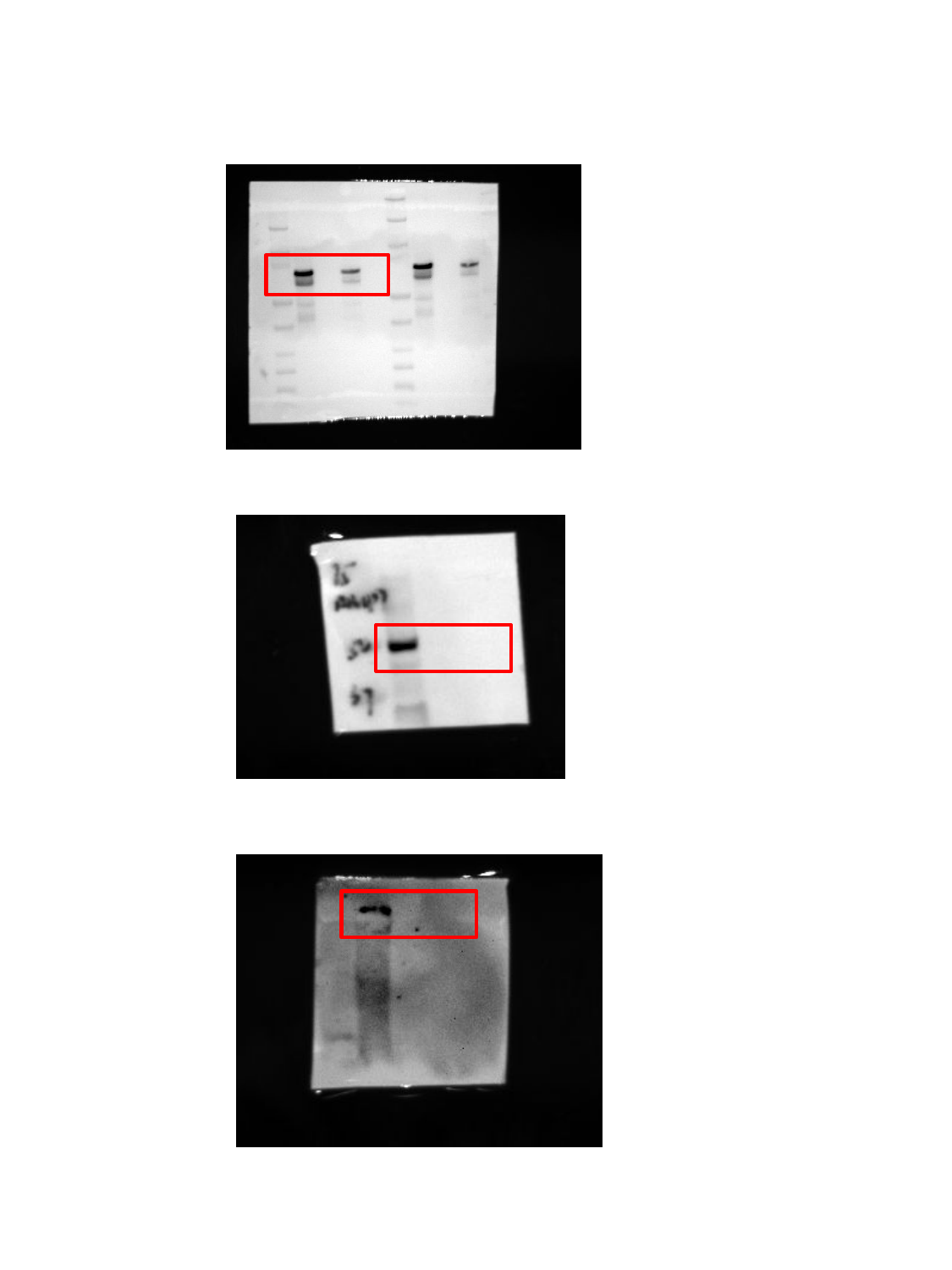

## Slide 18
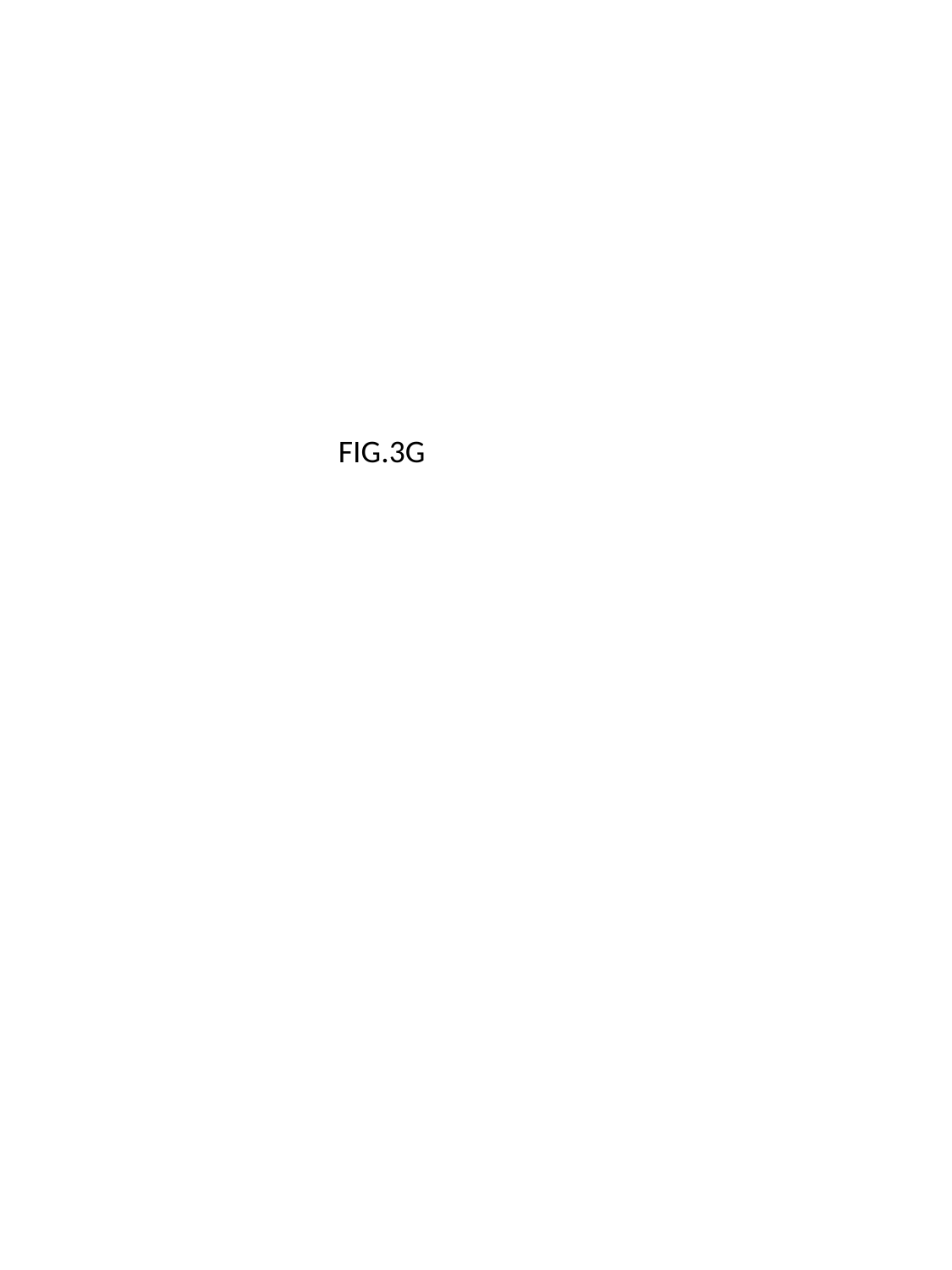

FIG.3G

## Slide 19
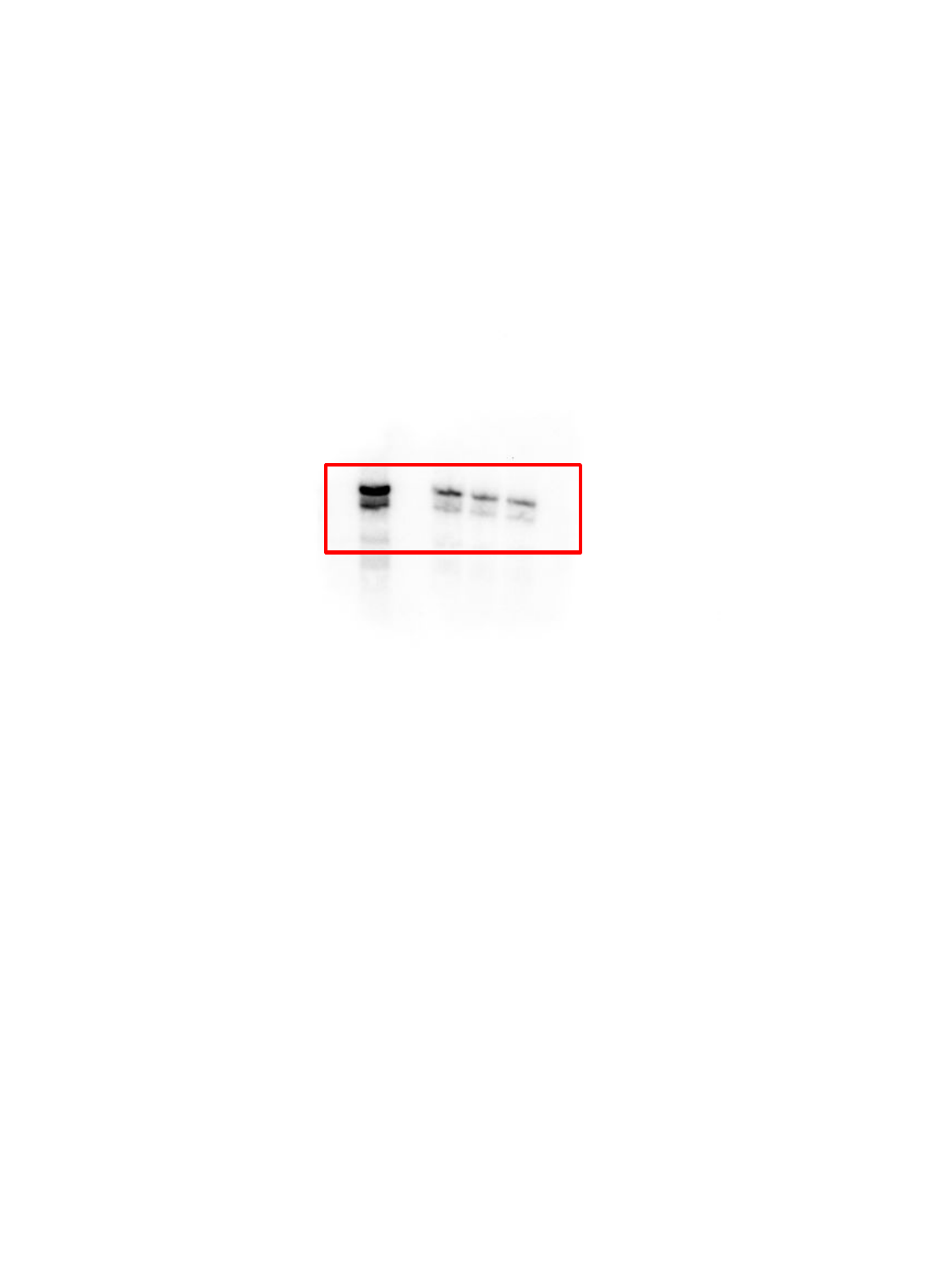

## Slide 20
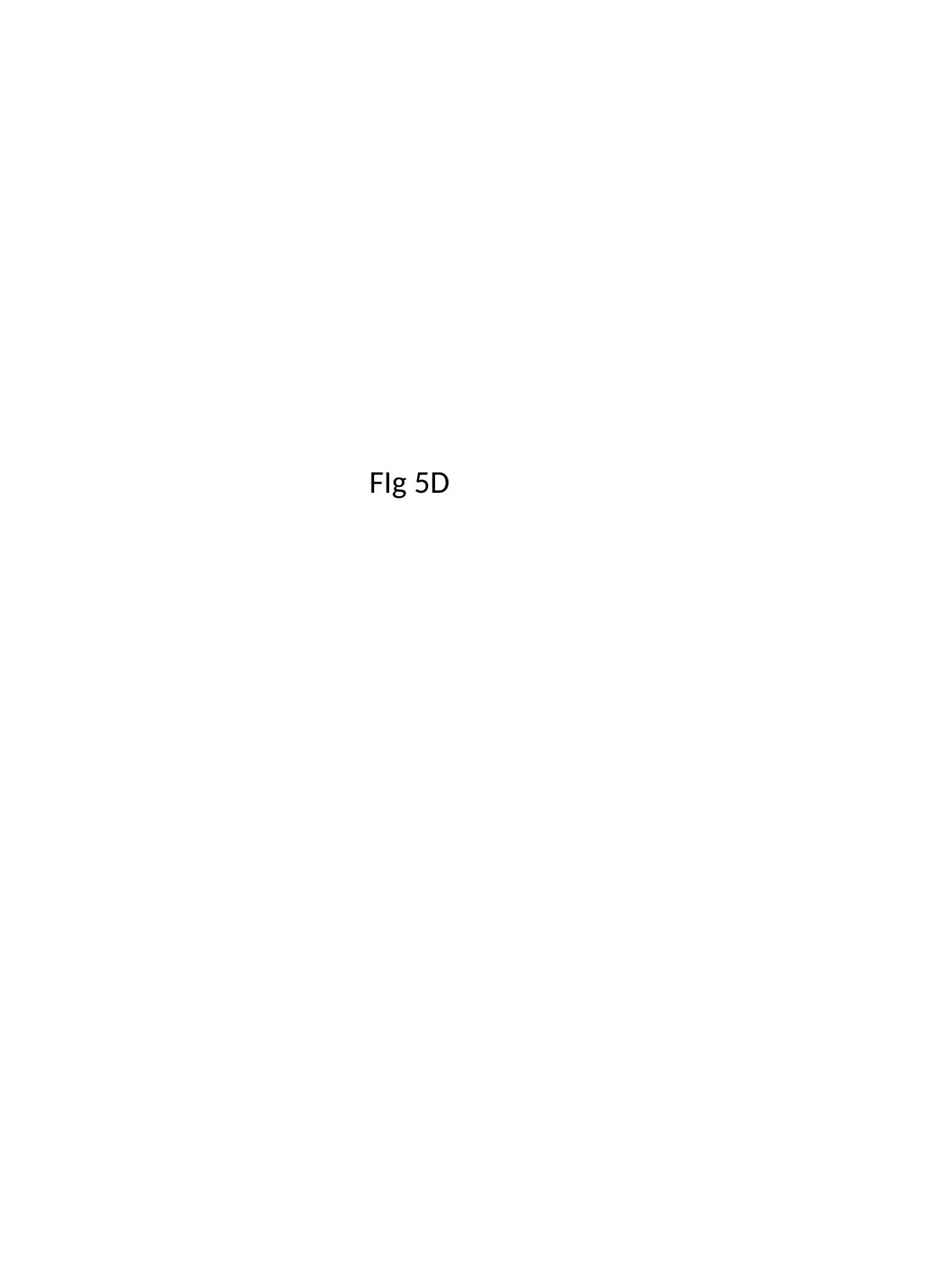

FIg 5D

## Slide 21
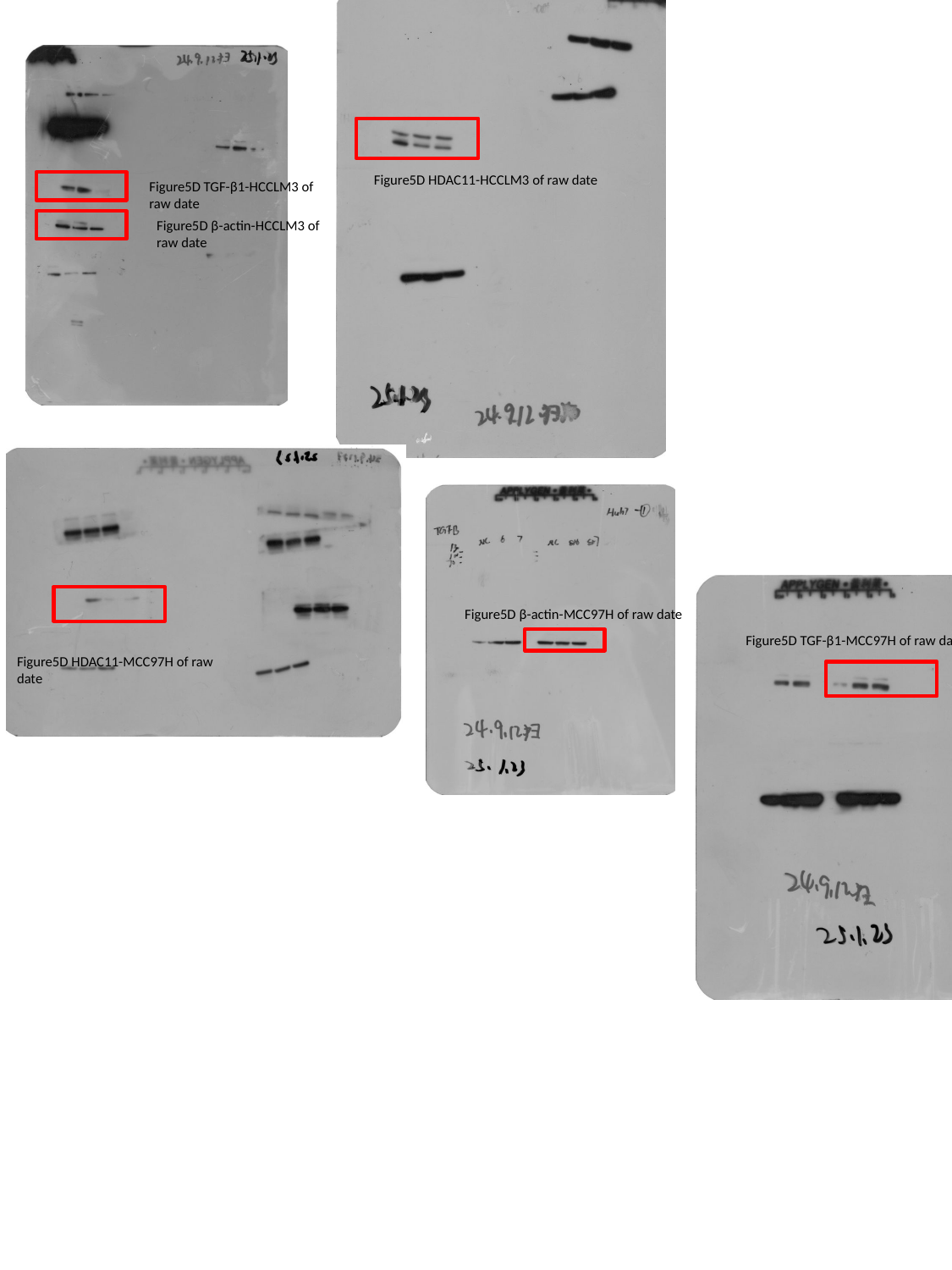

Figure5D HDAC11-HCCLM3 of raw date
Figure5D TGF-β1-HCCLM3 of raw date
Figure5D β-actin-HCCLM3 of raw date
Figure5D β-actin-MCC97H of raw date
Figure5D TGF-β1-MCC97H of raw date
Figure5D HDAC11-MCC97H of raw date

## Slide 22
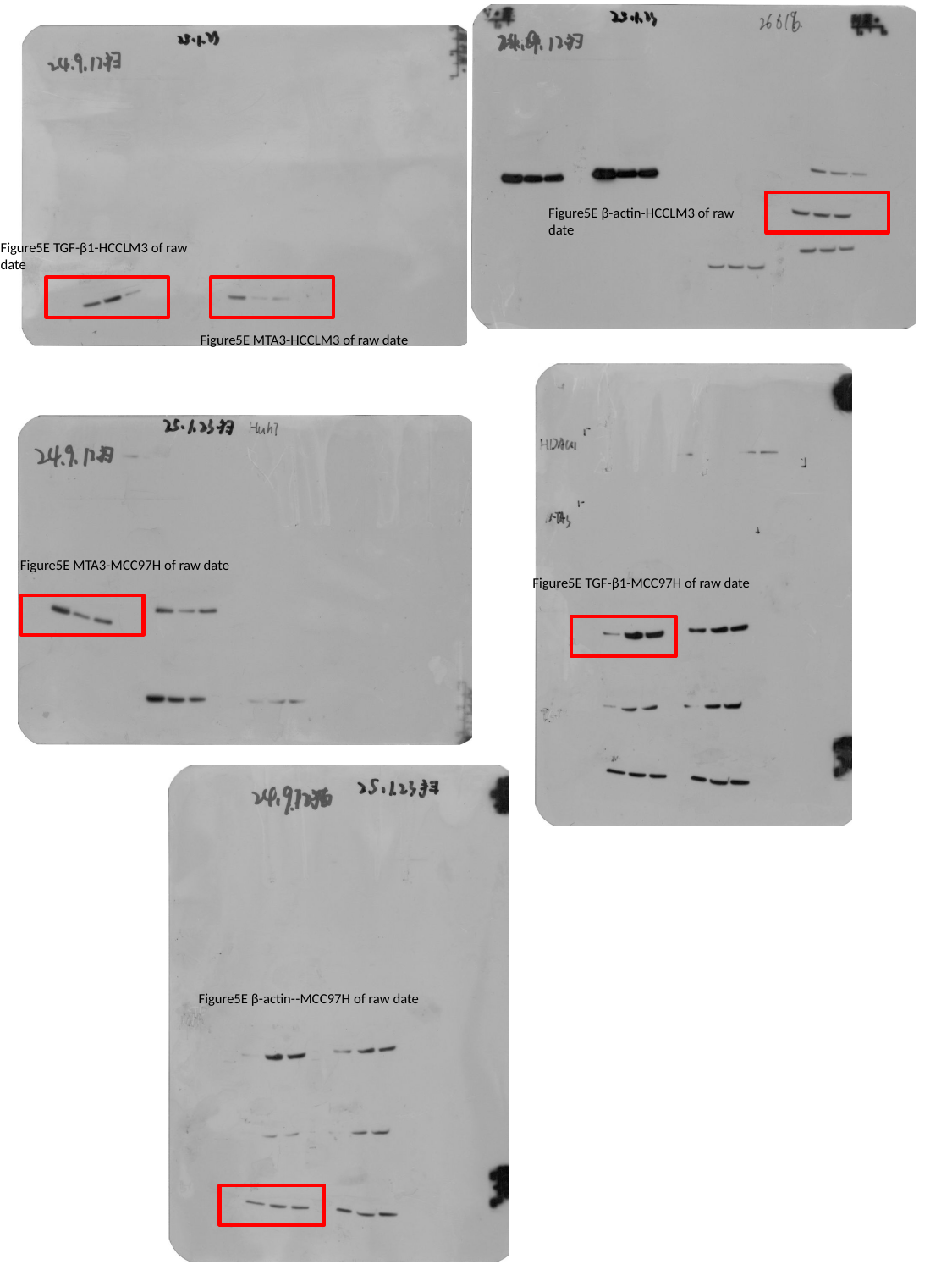

Figure5E β-actin-HCCLM3 of raw date
Figure5E TGF-β1-HCCLM3 of raw date
Figure5E MTA3-HCCLM3 of raw date
Figure5E MTA3-MCC97H of raw date
Figure5E TGF-β1-MCC97H of raw date
Figure5E β-actin--MCC97H of raw date

## Slide 23
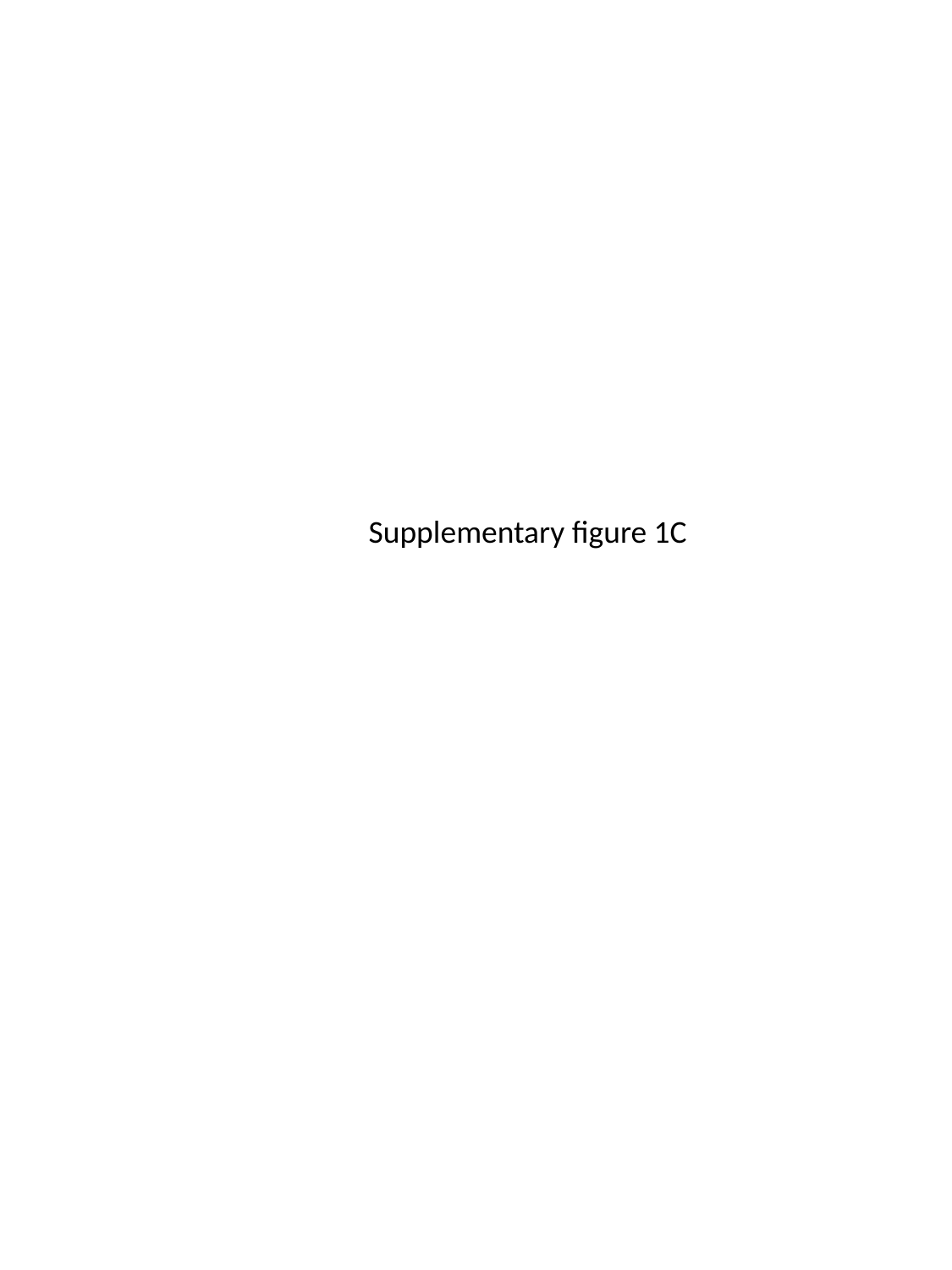

Supplementary figure 1C

## Slide 24
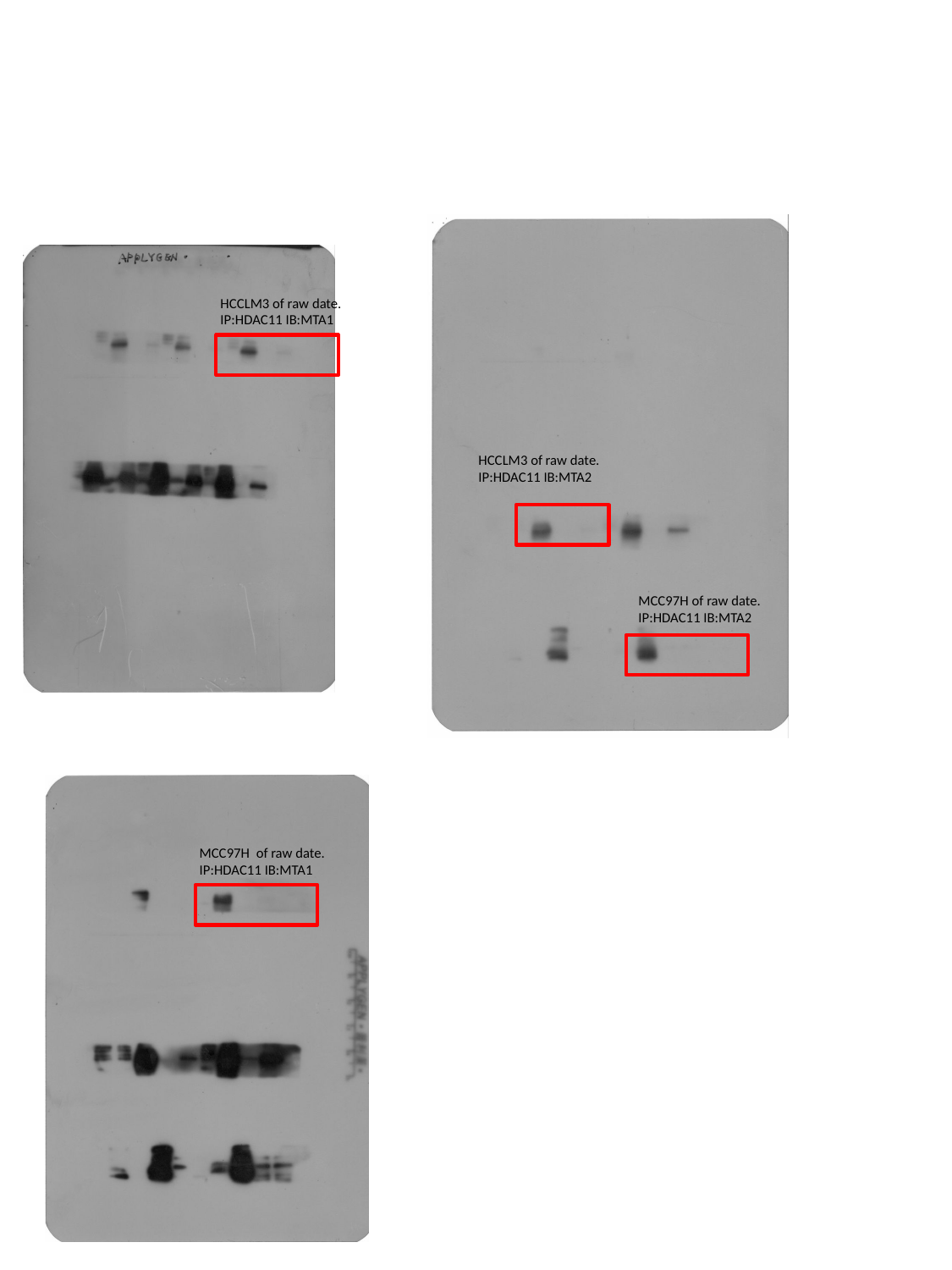

HCCLM3 of raw date.
IP:HDAC11 IB:MTA1
HCCLM3 of raw date.
IP:HDAC11 IB:MTA2
MCC97H of raw date.
IP:HDAC11 IB:MTA2
MCC97H of raw date.
IP:HDAC11 IB:MTA1
